# The cost-effectiveness of unilateral magnetic resonance-guided focused ultrasound in comparison with unilateral deep brain stimulation for the treatment of medically refractory essential tremor in England

**DOI:** 10.1259/bjr.20220137

**Published:** 2022-09-27

**Authors:** Ayesha Jameel, Anne Meiwald, Peter Bain, Neekhil Patel, Dipankar Nandi, Brynmor Jones, Georgie Weston, Elisabeth J Adams, Wladyslaw Gedroyc

**Affiliations:** ^1^ Department of Surgery and Cancer, Imperial College, London, United Kingdom; ^2^ Department of Radiology, Imperial College Healthcare NHS Trust, London, United Kingdom; ^3^ Division of Brain Sciences, Imperial College London, London, United Kingdom; ^4^ UK Aquarius Population Health Limited, London, United Kingdom; ^5^ Department of Neurosciences, Imperial College Healthcare NHS Trust, London, United Kingdom

## Abstract

**Objectives:**

This study aims to ascertain the cost-effectiveness of magnetic resonance-guided focused ultrasound (MRgFUS) for the treatment of medically refractory Essential Tremor (mrET) in England. Essential Tremor (ET) is the most common movement disorder affecting approximately 1 million in the UK causing considerable societal impact affecting patients, carers and the wider healthservice. Medical treatment has mixed efficacy, with approximately 25–55% of ET medication refractory. Deep brain stimulation (DBS) is a proven neurosurgical treatment; however, the risks of surgery and anaesthesia mean some patients are ineligible. MRgFUS is an emerging noninvasive technique that causes tremor suppression by thermal ablation of tremor-sensitive brain tissue. Several international clinical trials have demonstrated MRgFUS is safe and clinically effective; however, to-date no cost-effectiveness study has been performed in Europe.

**Methods:**

A Markov model was used to assess two subpopulations of mrET – those eligible and those ineligible for neurosurgery – in the context specific to England and its healthcare system. For those eligible for neurosurgery, MRgFUS was compared to DBS, the current standard treatment. For those ineligible for neurosurgery, MRgFUS was compared to treatment with medication alone. The model calculated the Incremental cost-effectiveness ratio (ICER) with appropriate sensitivity and scenario analyses.

**Results:**

For those eligible for neurosurgery: In the model base case, the MRgFUS was economically dominant compared to DBS; MRgFUS was less costly (£19,779 *vs* £62,348) and more effective generating 0.03 additional quality-adjusted life-years (QALYs) per patient (3.71 *vs* 3.68) over the 5-year time horizon.

For those ineligible for neurosurgery: In the model base case, MRgFUS cost over £16,000 per patient more than medication alone (£19,779 *vs* £62,348) but yielded 0.77 additional QALYs per patient(3.71 *vs* 2.95), producing an incremental cost-effectiveness ratio (ICER) of £20,851 per QALY. This ICER of £20,851 per QALY falls within the National Institute for Clinical Excellence’s (NICE) willingness to pay threshold (WTP) of 20,000–30,000 demonstrating the cost-effectiveness profile of MRgFUS.

**Conclusion:**

This study demonstrates the favourable cost-effectiveness profile of MRgFUS for the treatment of mrET in England; in both patients suitable and not suitable for neurosurgery.

**Advances in knowledge:**

The introduction of MRgFUS as a widely available ET treatment in UK is currently undergoing the necessary stages of regulatory approval. As the first European study, these favourable cost-effectiveness outcomes (notably the model base case ICER falling within NICE’s WTP) can provide a basis for future commissioning of brain MRgFUS treatments in the UK, Europe and globally.

## Introduction

Essential Tremor (ET) is a progressive neurological disorder characterised by bilateral arm tremor, that can extend to the body.^1^ Numerous studies have demonstrated the impact of manual impairment on activities of daily living, often leading to disability, social handicap and increased risk of depression and anxiety.^2,3^ Importantly, tremor in ET occurs in the absence of other progressive neurological processes, *e.g*. sensory, motor or mood disturbance; this is a key distinction from other conditions which may include tremor such as Parkinson’s disease.^1^ As the most prevalent movement disorder worldwide (~7 million in the USA^4^; ~1 million in the UK^5^), the societal impact from ET disability is considerable.^3,6,7^

The pathophysiology of ET remains under debate; theories include disordered stimulation of the cerebello-thalamo-cortical network.^8–10^ Medical treatments including propanol and primidone have mixed efficacy. Patients may trial combination therapy, although many experience waning clinical effectiveness^11^ with approximately 25–55% patients medication refractory.^6^ Deep Brain Stimulation (DBS), where electrodes connected to a stimulator are implanted deep in the brain, is a proven surgical treatment for ET.^11,12^ However, its clinical effectiveness can wane over time and most patients continue to take tremor medication.^13^ DBS patients require regular follow-up for stimulator optimisation and some complications including lead fracture, infection and battery failure can require re-operation.

Magnetic resonance-guided focused ultrasound (MRgFUS) is an emerging technique to treat various neurological conditions including ET.^14^ MRgFUS focuses multiple high-energy, low-frequency ultrasound beams on a single tremor-specific site. Heat generated at this site leads to thermal coagulative necrosis of the tremor-specific tissue^15^ monitored in real-time by magnetic resonance thermography. This non-invasive technique does not require general anaesthesia, making it suitable for patients ineligible for neurosurgery. As the patient is awake, treatments are tailored to individual neuroanatomy with precision targeting of tremor sensitive tissue, thus sparing non-tremor sensitive tissue, and minimising adverse effects. Several international^16,17^ and national^18^ clinical trials have demonstrated MRgFUS a safe and clinically effective treatment for ET.

Depending on individual healthcare systems, commissioning of new treatments and services are required to go through many stages of regulation and approval. In the USA, the first pilot study in 2013^19^ paved the way for FDA approval in 2016 and state-wide Medicare approval in 2020. In the UK, research and private treatments have been performed at Imperial College Healthcare Trust since 2016. National Health Service (NHS) England has now commissioned 150 ET treatments per year at designated centres.

All healthcare systems are required to consider how they allocate scarce resources. For both smaller private providers and large government systems, limited funds need to be allocated for the management of both acute and chronic disease. With persistent competition for funding, many institutions set in place processes, guidelines and minimum evidential requirements before decisions on health policy or funding can be made. In the UK, the National Institute of Clinical Effectiveness (NICE) is instrumental in decision making on health policy.^20^ NICE set parameters and recommendations upon which NHS England, Wales and Northern Ireland will often base national, regional and local healthcare commissioning. However, the impact of NICE’s recommendations are far reaching because their reviews are publicly available and readily generalisable to other centrally funded healthcare systems thus a positive or negative decision by NICE can have market implications beyond the UK.^21^

When considering novel or emerging treatments, NICE compares the new healthcare intervention (*e.g.,* MRgFUS) to an existing treatment or standard of care (*e.g.,* DBS) including cost and clinical effectiveness^22^ with particular regard to the outcomes of health technology assessments that demonstrate the Incremental Cost Effectiveness ratio (ICER) of the new intervention.^22^ To date, no cost-effectiveness study has been performed in the UK or Europe to assess MRgFUS in the treatment of ET.

### Aims

This study assesses the cost-effectiveness of MRgFUS treatment for medically refractory Essential Tremor (mrET) in England; comparing MRgFUS treatment to both the current standard for those eligible for neurosurgical treatment (DBS with medication) and those ineligible for neurosurgical treatment (medication alone). The study considers the clinical effectiveness, safety profile, costing implications and the impact of quality of life for each treatment option in England.

## Methods and materials

### Model structure

A Markov cohort model was developed to estimate the cost-effectiveness of MRgFUS in England as a treatment for moderate to severe mrET in England. A Markov cohort model is commonly used for cost-effective analyses, following a cohort of patients through different health states over time; transitions between these health states are defined by a probability.^23^ This model was adapted from a model exploring the cost-effectiveness of MRgFUS in Canada^24,25^; the structure and inputs were refined to ensure the approach was appropriate for an English population and the perspective of NHS England.

**Figure 1.**
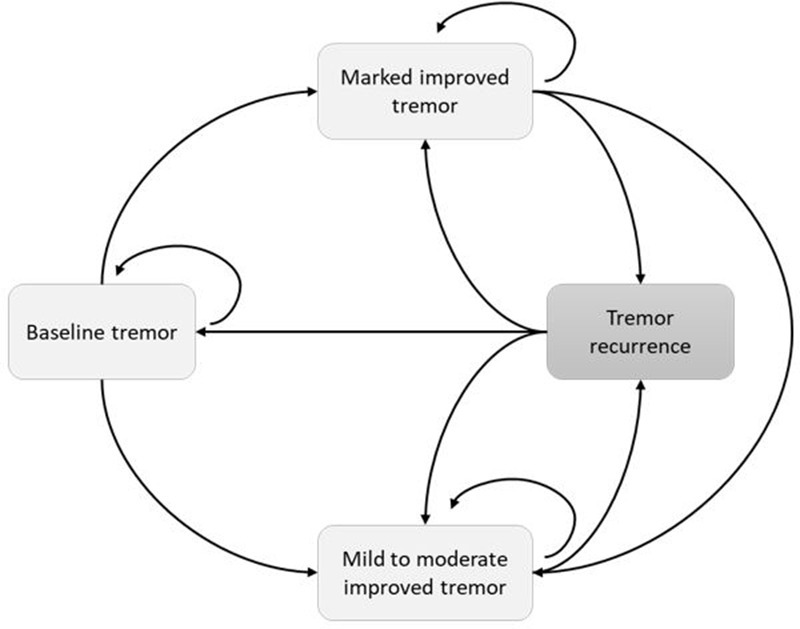
Markov model outlining the health states patients can move into post-procedure (DBS and MRgFUS).

The model assessed MRgFUS in two subpopulations of mrET patients: mrET patients who are suitable for DBS and mrET patients who are not suitable for DBS (Figure 1). The two interventional strategies (DBS and MRgFUS) had the same model structure and used a cycle length of 1 year. The model approach is described in Table 1 with further detail provided in Appendix 1.

Supplementary Table 1.

**Table 1.** Outlining the model approach, probability inputs, costing inputs, utility and disutility values. Further costing detail is provided in the supplementary information (Table S3-7; Appendix 3).

*Model approach*	*Model Population*	Inclusion criteria Moderate to severe mrET defined as a postural or intention tremor score of ≥2 on the Clinical Rating Scale for Tremor (CRST).^26^ Minimum tremor-related disability cut off score of ≥2 on the disability subsection (Part C) of the CRST.^27^ Exclusion criteria Previous intervention for tremor *e.g*. DBS or MRgFUS. Population Number The number of patients in England with mrET was estimated using several assumptions (Table S1Table S1).
	*Time horizon*	5 year time horizon Incorporates expected differences between strategies *e.g*. Tremor recurrence, long-term adverse events No long-term data for MRgFUS past 5 years^28^
	*Discount rate*	3.5% discount rate was applied to costs and utilities in the model in line with NICE guidelines.^29^
	*Perspective*	Cost-utility analysis performed from the perspective of NHS England.
	*Construction*	Microsoft excel (Microsoft corporation, Redmond, Washington dc, USA).
	*Health states modelled*	Four health states modelled Baseline tremor (BT), Mild to Moderately improved tremor (MMT) Marked improved Tremor (MT) Tremor recurrence (TR).
	*Adverse events*	Affect quality of life and/or incur healthcare costs. Only included if they required physician input or had a large impact on quality of life (Table S2). Transient defined <1year post procedure Long term defined>1 year post procedure
	*Mortality*	Assumptions included (*literature referenced):* No additional excess mortality from mrET nor procedures (DBS or MRgFUS)^16^ At any point during the model time frame a patient may die from all-cause mortality
*Probability Inputs*	*Improvement post-procedure*	Assumptions included (*literature referenced):* MRgFUS and DBS have the same clinical effectiveness but with different adverse event profiles.^30–32^ Treatment benefit for MRgFUS is immediate.^15^ Treatment benefit for DBS starts at 3 months due to device optimisation (validated by clinical experts). MMT and MT proportions based on the English clinical trial^18^ with data augmentation using the Wilson Method (Appendix 1). Unsuccessful procedure considered less than 10% improvement, patients remained in BT health state.
	*Tremor Severity*	Assumptions included (*literature referenced):* MrET stable over the time horizon, although ET is a naturally progressive disease. In the model patients only deteriorated due to tremor recurrence or waning procedural effectiveness.
	*Tremor recurrence*	Assumptions included (*literature referenced):* For DBS, the probability of tremor recurrence (defined in the study as a pattern of tolerance) is 4%.^20^ For MRgFUS, the probability of recurrence is 8.3%.^18^ Recurrence rates were converted to an annual probability in the model assuming a constant probability over time.
	*Re-operation after tremor recurrence*	Assumptions included (*literature referenced):* Re-operation resulted in the same outcomes as the primary procedure.^25^ When TR patients undergo re-operation they receive the same surgical procedure as their first procedure.
*Cost Inputs*	*Medication*	Assumptions included (*literature referenced):* All patients have the same ET medications costs independent of which health state they were in. Medication will continue to be taken despite a MT health state due to tremor in the untreated arm.
	*DBS*	Estimated using NHS reimbursement costs^33^ and validated by clinical experts (Table S3). This cost included all pre-, peri- and post-procedure and follow-up costs for the first year.
	*MRgFUS*	From the NHS England reimbursement costs provided by Imperial College Healthcare NHS Trust included all pre-, peri-, post-procedure and follow-up costs for the first 5 years. The model assumes there are no additional costs associated with the transient or permanent adverse events for MRgFUS as these are managed during routine follow-up.
*Utility values*	*Baseline Tremor (BT):*	Based on Herceg *et al.,* 2012^34^ as per Canadian Model^24^
	*Mild Tremor Improved (MT*)	Based on Herceg *et al.,* 2012^34^ as per Canadian Model^24^
	*Marked to Moderate Tremor Improvement (MMT):*	The utility was assumed to be an average between BT and MT
	*Tremor Recurrence (TR):*	The utility of TR was assumed to be the same as BT.
*Disutility values*	*Reoperation*	Assumptions included: No additional disutility associated with re-operation Any adverse events were captured in the same manner as for the first procedure.
	*Adverse Events*	To include decreases in quality of life due to the transient and permanent adverse events, a weighted disutility was calculated using this four-step process outlined in appendix 1.

The model included patients with moderate to severe mrET without previous intervention, *i.e*. patients undergoing DBS or MRgFUS for the first time. MrET was defined by a postural or intention tremor score of ≥2 on the Clinical Rating Scale for Tremor (CRST)^26^ and a minimum tremor-related disability cut off score of ≥2 on the disability subsection (Part C) of the CRST.^27^

Four health states were included in the model: baseline tremor (BT), mild to moderately improved tremor (MMT), marked improved tremor (MT), and tremor recurrence (TR).

- The BT state includes patients who live with disabling mrET if they do not undergo intervention or if they have an unsuccessful procedure (DBS or MRgFUS) defined as less than 10% improvement in their CRST score.
- The MMT and MT states include patients who have 10–50% improvement and 50–100% improvement in their CRST score respectively.^24,25^
- The TR is a transient one cycle state for patients who experience tremor recurrence after an intervention that requires re-operation.

### Outcomes

Model outcomes were the total costs per patient (2019 GBP) and quality-adjusted life-years (QALYs) for each strategy over a 5-year time horizon based on the available temporal data for MRgFUS. QALYs are a measure of disease burden based on quantity and quality of life lived, commonly used in health outcomes analyses.^35^ The Incremental Cost-Effectiveness Ratio (ICER) is the cost per QALY gained when an intervention is introduced, which was calculated for MRgFUS versus DBS and for MRgFUS versus no procedure ((cost per MRgFUS – comparator) / (cost of MRgFUS – comparator)). A discount rate of 3.5% was applied to costs and utilities in the model in line with NICE guidelines.^29^

### Model inputs

The model parameters are described in Table 2. The base case model included mrET patients starting at age 70 years in England. ET affects males and females equally,^37^ thus the proportion of males is based on Ofiice for National Statistics (ONS) data.^38^ There are an estimated 26,202 patients with ET, of which 1,769 had moderate to severe mrET, and of those, 1,415 were eligible for DBS. Details are in Table S1; Appendix 3 ^24,38–41^.

**Table 2.** Model parameters Further detail is provided in the supplementary information (Table S1-7; Appendix 3)

Parameter	Value	Source
Clinical effectiveness		
Proportion with marked improvement post-procedure	88.9%	^18^
Proportion with mild to moderate improvement post-procedure	10.0%	^18^
Proportion with no improvement post-procedure	1.1%	^18^
Annual proportion moving from marked to mild due to waning in surgical effectiveness (MRgFUS)	9.2%	^18^
Annual proportion moving from marked to mild due to waning in surgical effectiveness (DBS)	7.7%	^36^
Annual probability of recurrence in (MRgFUS)	1%	^18^
Annual probability of recurrence in (DBS)	3.9%	^36^
Annual probability of re-operation after recurrence	5%	Assumption (based on expert opinion)
Costs		
DBS procedure (all resource use for 1 year)	£47,627	Table S3
MRgFUS procedure (all resource use for 5 years)	£16,500	Assumption (based on expert opinion)
Medication cost per year	£744	Table S3
*Ongoing monitoring costs*		
No procedure	£169	Table S6
DBS 2 + years	£3,172	Table S6
MRgFUS 5 + years	£169	Table S6
*Adverse events costs*		
Infection	£657	Table S7
Intracranial haemorrhage	£20,545	Table S7
Lead fracture or migration	£14,777	Table S7
Gait disturbance	£0	Table S7
Speech problem	£42	Table S7
Utilities		
Alive with disabling tremor (baseline)	0.69	^34^
Improved tremor post-procedure (marked improvement – no adverse events)	0.91	^34^
Improved tremor post-procedure (mild to moderate improvement – no adverse events)	0.80	Assumed to be between baseline and marked improvement post-procedure
Improved tremor post-procedure with adverse events (marked improvement) (MRgFUS) Short term (year 1) Long term (year 2+)	0.91 0.90	A weighted average utility including the disutility of common adverse events using the adverse event proportion found in Table S2 and corresponding utilities in Table S8.
Improved tremor post-procedure with adverse events (marked improvement) (DBS) Short term (year 1) Long term (year 2+)	0.91 0.91	A weighted average utility including the disutility of common adverse events using the adverse event proportion found in Table S7 and corresponding utilities in Table S8.
Improved tremor post-procedure with adverse events (mild/moderate improvement) (MRgFUS) Short term (year 1) Long term (year 2+)	0.80 0.79	A weighted average utility including of the disutility of common adverse events using the adverse event proportion found in Table S7 and corresponding utilities in Table S8.
Improved tremor post-procedure with adverse events (mild/moderate improvement) (DBS) Short term (year 1) Long term (year 2+)	0.80 0.76	A weighted average utility including of the disutility of common adverse events using the adverse event proportion found in Table S2 and corresponding utilities in Table S8.
Others		
Population	1,415	Calculated using assumptions found in Table S1
DBS onset of benefit (months)	3 months	Assumption (based on expert opinion)

DBS, deep brain stimulation;MRgFUS, magnetic resonance-guided focused ultrasound.

a recurrence refers to diminished tremor control back to baseline

The proportion of the mrET population moving to each health state at each cycle was based on predicted population behaviour and response to treatment from the English Clinical Trial data^42^ (Table 1).

The costs were estimated from the perspective of NHS England and were reported in 2019 GBP. We included the following costs: surgical procedure, medication for ET, ongoing patient monitoring, and management of adverse effects (Table 1; details in Appendix 3 (Table S3-7; Appendix 3)). The DBS procedure cost was estimated using NHS reimbursement costs^33^ and validated by clinical experts (Appendix 3 Supplementary Table S3). The cost included all pre-, peri- and post-procedure and follow-up costs for the first year including all imaging and clinician time. For MRgFUS, the NHS England reimbursement cost was provided by Imperial College Healthcare NHS Trust included all pre-, peri-, post-procedure and follow-up costs for the first 5 years. The model assumes there are no costs associated with the transient or permanent adverse events for MRgFUS as they are managed during normal follow-up. All patients were assumed to have the same ET medications costs independent of which health state they were in. Costing details can be found in the Appendix 3 Supplementary Tables S3-7.

The approach to utility values is outlined in Table 1. Values included the utility in each health state, and the disutility incurred from treatment-related adverse events.

### Uncertainty analyses

To explore the robustness of the base case results (derived from the baseline model inputs), uncertainty analyses were performed. A deterministic sensitivity analysis (DSA) and probabilistic sensitivity analyses (PSA) were performed to determine which parameters had the largest effect on model outcomes.

In the DSA, the input values for probabilities, utilities and costs were varied between their assigned upper and lower limits (either the 95% confidence interval (CI) from baseline or by 20% of the original values (Table S8; Appendix 3)). Only the results for those parameters that gave >5% difference from the base case, either using the high or low value, were reported.

A Monte Carlo PSA was run with 1,000 iterations. Each input value was independently sampled from their assigned distribution (based on data from the literature or estimated distributions). Probabilities were assigned a β distribution and cost parameters were assigned a γ distribution (Table S8; Appendix 3). All inputs shared by the two strategies (MRgFUS and DBS) were assigned the same value for that given iteration; all other inputs were varied independently between the strategies. The probability of MRgFUS being cost-effective at different Willingness to Pay Thresholds (WTP) (*i.e.,* different maximum values for an acceptable cost per gained QALY) was reported as a cost-effectiveness acceptability curve.

Five scenario analyses were run: 1) varying the starting age of patients in the model to age 40, 50 and 60; 2) varying the time-horizon; 3) modelling current care (*i.e.* a blend of patients who have no procedure and those that undergo the DBS procedure); 4) no discounting of costs and QALYs; and 5) using inputs from a North American Randomised Control Trial (RCT) to validate the English results.^16^ For scenario five sensitivity analyses (DSA, PSA) were performed again.

## Results

For the sub-population of mrET patients eligible for DBS, the model demonstrated the favourable cost-effectiveness of MRgFUS compared to DBS. MRgFUS was less costly (£19,779 *vs* £62,348) and more effective generating 0.03 additional QALYs (3.71 *vs* 3.68) per patient over the 5-year time horizon (Table 3). Thus MRgFUS is economically dominant compared to DBS.

**Table 3.** The baseline results for the outcomes (MRgFUS versus all comparators) per patient; 5-year horizon

Strategy	Costs (£)	Δ^1^ Cost (£)	QALY	Δ^1^ QALY	ICER (£/QALY)
Base case (England data)					
MRgFUS	19,779		3.71		
No procedure	3,735	16,044	2.95	0.77	**20,851**
DBS	62,348	−42,569	3.68	0.03	**MRgFUS is dominant**
Scenario based on RCT data					
MRgFUS	19,772		3.52		
No procedure	3,735	16,037	2.95	0.57	**27,959**
DBS	61,531	−41,759	3.44	0.08	**MRgFUS is dominant**

The change in costs and QALYs is the comparator strategy always compared to the MRgFUS strategy *i.e.,* the average change and ICER and not estimating the incremental gains. Difference and total costs values are reported to the nearest integer. Baseline results are with discounting. MRgFUS, magnetic resonance-guided focused ultrasound; DBS, deep brain stimulation; ICER, incremental cost-effectiveness ratio; QALY, Quality Adjusted Life Year

^1^Δ = the difference in cost or QALY (MRgFUS – comparator strategy). A strategy is termed as dominant when it is both cost-saving (*i.e.,* a negative incremental cost) and more effective (*i.e.,* a positive incremental QALYs).

In patients not suitable for DBS, the MRgFUS strategy cost over £16,000 more than medication alone (£19,779 *vs* £3,735) but yielded 0.77 additional QALYs (3.71 *vs* 2.95) per patient, producing an ICER of £20,851 per QALY gained over a 5-year horizon. This ICER falls within the NICE willingness to pay threshold of £20,000–30,000 per QALY and thus demonstrates the likely cost-effectiveness of unilateral MRgFUS for the treatment of mrET.

### Deterministic sensitivity analysis (DSA)

**Figure 2.**
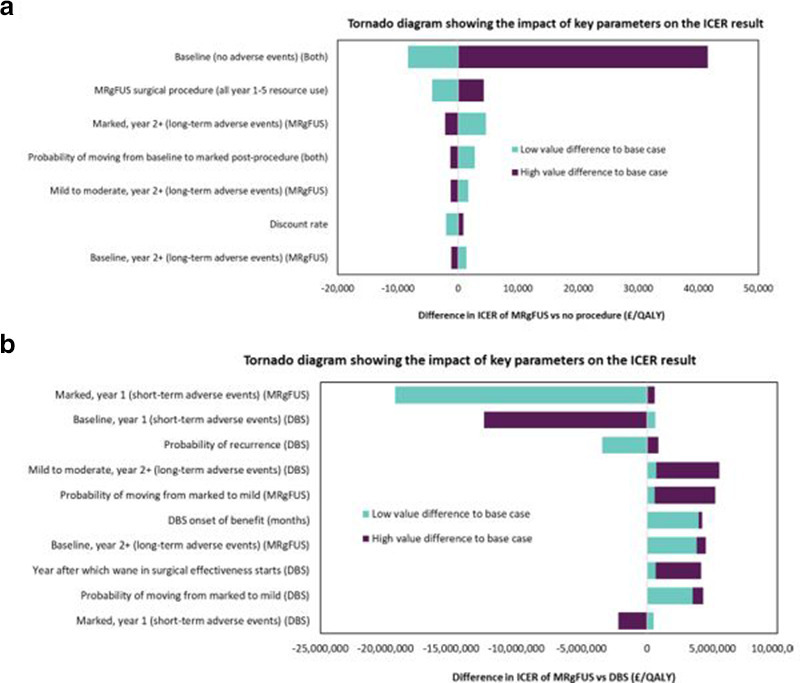
*Results of the deterministic sensitivity analysis for A) MRgFUS vs No Procedure B) MRgFUS vs DBS: the impact on the ICER of varying each parameter individually to the high and low values in a one-way sensitivity analysis*.

The impact of varying the model inputs on the results is presented in a tornado graph (Figure 2) showing the difference between the MRgFUS and DBS strategies.

DSA results for MRgFUS versus DBS demonstrated that when the input parameters were varied between their low and high values, several parameters had the biggest impact on the ICER: the utility for the marked improvement health state in the first year for those undergoing MRgFUS (-£20,561,429 to -£697,086), the utility for baseline state in the first year for those undergoing DBS (-£672,348 to -£13,748,994), and the probability of recurrence for those undergoing DBS (-£4,720,374 to -£418,743).

DSA for MRgFUS versus no procedure demonstrated these inputs impacted the ICER most: the baseline utility (£12,517–62,397), the cost of MRgFUS procedure (£16,535–25,167) and utility for the marked improvement health state in year two onwards for those undergoing MRgFUS (£25,504–18,691).

### Probabilistic sensitivity analysis (PSA)

**Figure 3.**
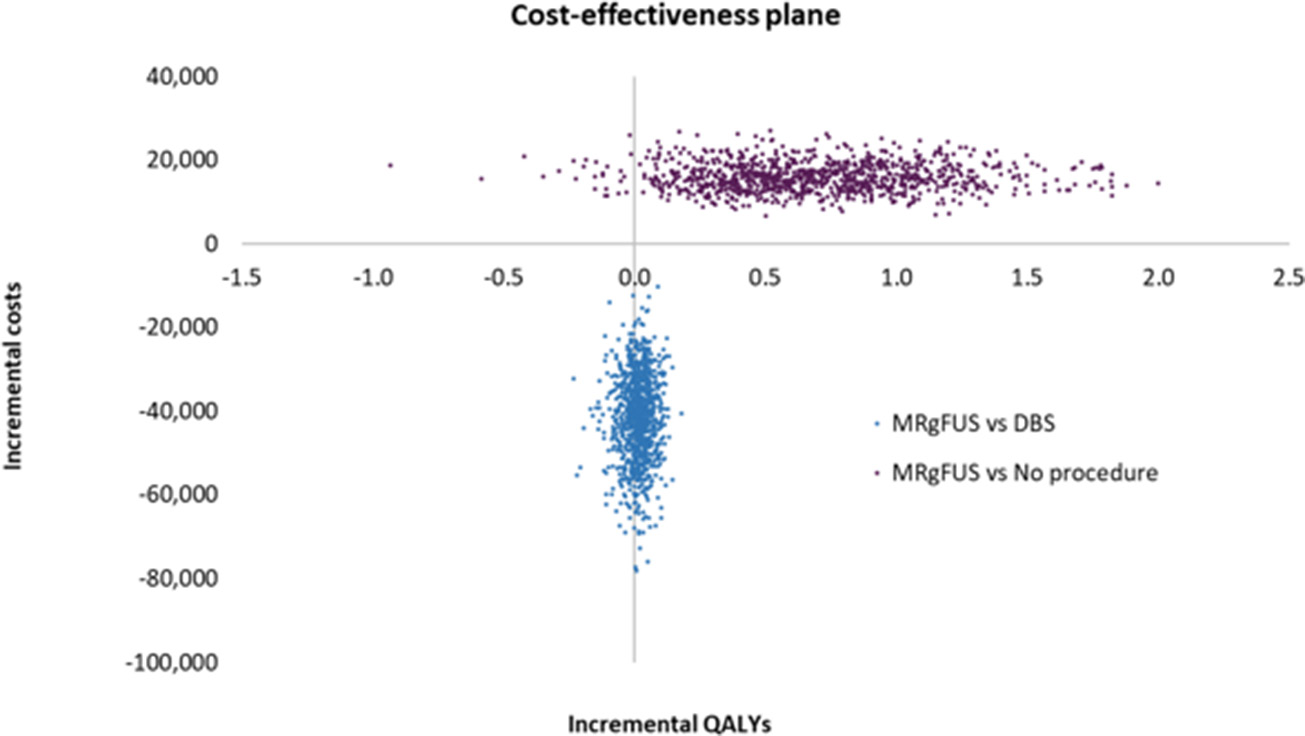
Result of probabilistic analysis for MRgFUS vs No Procedure and MRgFUS vs DBS: based on 1,000 iterations illustrating the distribution of the ICERs

**Figure 4.**
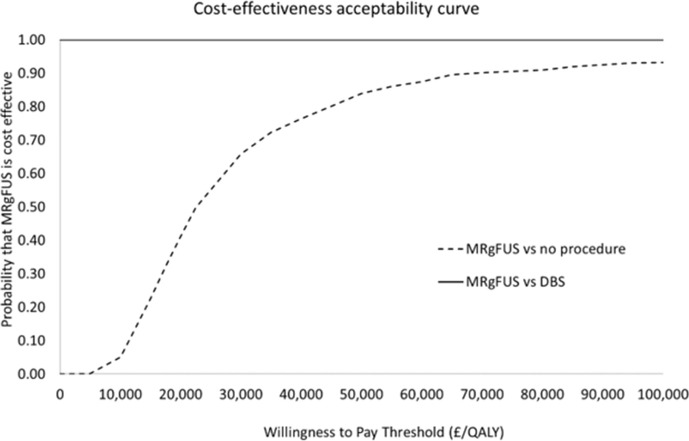
Cost-effectiveness acceptability curve for MRgFUS vs No Procedure and MRgFUS vs DBS: showing the probability that MRgFUS is cost-effective at different Willingness to Pay Thresholds for cost-effectiveness

The impact on the outcomes of varying the parameter inputs in the PSA is shown in Figure 3. The probability of MRgFUS being cost-effective at different WTPs is reported as a cost-effectiveness acceptability curve (Figure 4).

There was large uncertainty in the results for MRgFUS versus DBS. The average ICER was -£1,032,614 per QALY). The MRgFUS strategy was more cost-effective than DBS in 100% of the 1,000 iterations.

For MRgFUS versus no procedure, the ICER 95% CI varied between £15,194 to £202,391 (average £22,268). When using a WTP threshold of £30,000, the MRgFUS strategy was more cost-effective than no procedure in 66% of the 1,000 iterations and in under 2 of iterations were the differences in QALYs negative.

### Scenario analysis

When the starting age of patients entering the population was reduced (Table 4), the ICER remained dominant compared to DBS but there was little change in the incremental QALY (Table 4). Compared to no procedure, the ICER slightly reduced from £20,851 from the base case (aged 70) to £19,816 (aged 40).

**Table 4.** The results for the outcomes (MRgFUS versus all comparators, per patient); Age scenario (England data)

Age		Costs (£)	Δ^1^ Cost (£)	QALY	Δ^1^ QALY	ICER (£/QALY)
60	MRgFUS	19,892		3.84		
	No procedure	3,867	16,025	3.05	0.79	**20,175**
	DBS	62,914	−43,023	3.81	0.03	**MRgFUS is dominant**
50	MRgFUS	19,937		3.89		
	No procedure	3,920	16,017	3.09	0.80	**19,917**
	DBS	63,141	−43,204	3.86	0.03	**MRgFUS is dominant**
40	MRgFUS	19,955		3.91		
	No procedure	3,941	16,014	3.10	0.81	**19,816**
	DBS	63,231	−43,276	3.88	0.03	**MRgFUS is dominant**

The change in costs and QALYs is the comparator strategy always compared to the MRgFUS strategy *i.e.,* the average change and ICER and not estimating the incremental gains. Difference and total costs values are reported to the nearest integer. DBS, deep brain stimulation; MRgFUS, magnetic resonance-guided focused ultrasound; ICER, incremental cost-effectiveness ratio; QALY, Quality Adjusted Life Year

^1^Δ = the difference in cost or QALY (MRgFUS – comparator strategy). A strategy is termed as dominant when it is both cost-saving (*i.e.,* a negative incremental cost) and more effective (*i.e.,* a positive incremental QALYs).

The results were sensitive to the model time horizon (Table S10; Appendix 3). When a 1-year and 10-year time horizon were used MRgFUS was still the dominant strategy compared to DBS in the base case. Compared to no procedure, the ICER in the base case was £84,539 and £13,940 at the 1- and 10-year time horizon, respectively. The current care scenario showed that MRgFUS was always a cost-effective option, but it became the dominant strategy if at least 30% of the population was eligible for DBS (Table 5).

**Table 5.** The results for the outcomes per patient (assuming 100% of patients are suitable for MRgFUS versus a certain % of patients are not suitable for DBS); Current care scenario (England data)

Strategy	Patients eligible for DBS (%)	Costs (£)	Δ^1^ Cost (£)	QALY	Δ^1^ QALY	ICER (£/QALY)
MRgFUS		19,779		3.71		
Current care	0	3,735	16,044	2.95	0.77	**20,851**
	10	9,716	10,063	3.05	0.66	**14,214**
	20	15,670	4,109	3.07	0.64	**6,383**
	30	21,597	−1,818	3.14	0.58	**MRgFUS is dominant**
	40	27,498	−7,719	3.21	0.51	
	50	33,373	−13,594	3.28	0.44	
	60	39,221	−19,442	3.35	0.36	
	70	45,042	−25,263	3.43	0.28	
	80	50,837	−31,058	3.51	0.20	
	90	56,606	−36,827	3.60	0.12	
	100	62,348	−42,569	3.68	0.03	

Current care is a blend of patients who have no procedure and those that undergo the DBS procedure. The change in costs and QALYs is the comparator strategy always compared to the MRgFUS strategy *i.e.,* the average change and ICER and not estimating the incremental gains. Difference and total costs values are reported to the nearest integer. DBS, deep brain stimulation; MRgFUS, magnetic resonance-guided focused ultrasound; ICER, incremental cost-effectiveness ratio; QALY, Quality Adjusted Life Year

^1^Δ = the difference in cost or QALY (MRgFUS – comparator strategy). A strategy is termed as dominant when it is both cost-saving (*i.e.,* a negative incremental cost) and more effective (*i.e.,* a positive incremental QALYs).

Using the RCT inputs^16^ for the English model produced similar cost-effective outcomes as the original base case (Table 3). Further details on the RCT scenario results and sensitivity analyses are provided in Appendices 2, 3 (S10, S11, S12), 4 (F1-F3). A scenario was also run without discounting showing similar results (Appendix 3, S13).

## Discussion

This study demonstrates the favourable cost-effectiveness profile of unilateral MRgFUS for the treatment of mrET in England. For MRgFUS versus DBS, the MRgFUS strategy was economically dominant (less costly with 0.03 additional QALYs per patient) over a 5-year time horizon. Compared to no procedure (in patients not suitable for DBS) the MRgFUS strategy cost £16,000 more but yielded 0.77 additional QALYs per patient, producing an ICER of £20,851 per QALY gained, over a 5-year time horizon. The results were sensitive to analyses on assumptions regarding the time horizon, cost of MRgFUS, and utilities used.

### Health policy

In the UK, NICE typically recommends interventions with ICERs at £20,000–£30,000; this is considered their WTP threshold. Although not a rigid formula, interventions with ICERs below or within the WTP threshold are more likely to be commissioned than those with higher ICERs.^22^ The ICER of £20,851 per QALY gained falls within NICE’s WTP supporting the view that MRgFUS should be adopted into standard practice in the treatment of mrET.

This study demonstrates to health policymakers that MRgFUS is an advantageous curative treatment option for mrET which can reduce tremor, improve quality of life and be cost-effective. MRgFUS benefits patients as it is a non-invasive procedure with a favourable safety profile that can be performed as a day case without general anaesthetic. Patients have immediate results without the need for ongoing optimisation or follow up. MRgFUS can also benefit the healthcare system, as MRgFUS falls within acceptable WTP levels it can save costs and/or inform rational allocation of resources. As the model (including its assumptions and inputs) were from the perspective of NHS England, its outcomes may be helpful to other similarly publicly funded healthcare systems.

### Limitations

The study limitations centered mainly on model inputs and assumptions. Inputs were selected by a combination of literature review, known costs, predicted and estimated costs, expert opinion and/or a combination of all. The robustness of these inputs were constrained by the quality of available data on mrET, MRgFUS and DBS. Where there was incomplete or no data, assumptions were needed. Where possible these assumptions were validated by clinical experts. For example, MRgFUS and DBS were assumed to be equally clinically effective although no head-to-head clinical trials have been performed. Of note, the scenario and sensitivity analyses tested the robustness of these assumptions and consistently determined MRgFUS to be cost-effective. Some key limitations and assumptions are described here.

As the model was designed for the NHS England perspective, English data inputs were utilised in the base case. However, as the English clinical trial was a small-scale study, the results were compared to using data inputs from a larger scale MRgFUS Randomised Control Trial^43^; this produced similar cost-effective outcomes as the original base case, and hence supported the robustness of results.

The model assumed that all patients suitable for DBS are equally suitable for MRgFUS. Although there may be some patients whose skull density ratio makes them ineligible for MRgFUS. This is a very low proportion of patients that is unlikely to make a difference to the model outcomes.

As there are few longitudinal studies for MRgFUS or DBS, any assessment on the long-term efficacy of treatment is challenging thus the model was not extrapolated over a patient’s lifetime. The battery used in England has a longer life than the model time horizon,^44^ therefore costs did not include battery change for DBS however this would be a significant cost if included (Table S4, Appendix 3).

There are few studies reporting utility values for ET or mrET, thus utility values were taken from other neurological diseases. These assumptions are imperfect but provided insight into the disutility of chronic progressive disease.

Patient preference was assumed to be equal between MRgFUS and DBS. However, depending on their level of disability patients may have difficulty attending numerous follow-up appointments for DBS or may be unwilling to accept the risks associated with invasive surgery. MRgFUS may be preferable to patients *ceteris paribus*.

### Comparison with other MRgFUS cost-effectiveness studies

Results from this study are comparable with other international MRgFUS cost-effectiveness studies. Similar to this study, a US study found MRgFUS to be less costly and more clinically effective than DBS. A Japanese study looked at costs alone and also found MRgFUS to be cheaper than DBS.^45^ However, these Japanese and American studies did not ascertain ICERs.^46,47^ The ICERs and outcomes for the Canadian study were similar to this study.^24^

In the US study, the cost and the clinical effectiveness were calculated individually “measured by amount of utility added by the procedure”.^46^ The effectiveness value for MRgFUS was 0.194 *vs* 0.134 for DBS. DBS cost more (£22,450; converted 2016 US$27,906) than MRgFUS (£16,567; converted 2016 US $20,593). In the 2018 Canadian study,^48^ MRgFUS compared to no procedure had an ICER of £27,636 (converted from 2017 CAN$45,817) and MRgFUS was cheaper than DBS but less effective (ICER DBS versus MRgFUS of £78,927 (converted 2017 CAN$130,850)). The 2019 Japanese cost minimisation study (no quality of life estimate) was performed over 1 year.^47^ MRgFUS was determined cost-saving (£2,892; converted 2018 JPY400,380); the total costs per procedure for MRgFUS £15,494 (converted 2018 JPY2,145,037) and unilateral DBS were £18,386 (converted from 2018 JPY2,545,417).

### Future research

As the main challenge to estimating the cost-effevtieness was a lack of data, further research would benefit from improved data inputs. This could be attained through head to head RCT trials of MRgFUS and DBS, more longitudinal studies of MRgFUS and DBS considering long term efficacy, and ascertaining the utilities for mrET and patient treatment preferences. The DSA demonstrated particular sensitivity to utility values for both MRgFUS and DBS, dedicated studies ascertaining these inputs would improve any future cost-effectiveness analyses.

The model compared unilateral MRgFUS to unilateral DBS, ignoring the possibility of bilateral DBS treatments. Although there have been successful bilateral treatments for mrET using MRgFUS^49–51^ it is not yet standard of clinical practice in the UK and has not yet been commissioned by NHS England. Future research could compare bilateral MRgFUS to bilateral DBS.

## Conclusion

This study demonstrates the favourable cost-effectiveness of MRgFUS for the treatment of mrET in England; in patients who are suitable and those who are not suitable for DBS, MRgFUS was deemed cost-effective. As the first European study, outcomes from this study can provide a basis for future commissioning of brain MRgFUS treatments in the UK, Europe and worldwide. MRgFUS can benefit patients and the healthcare systems and should be considered as a treatment option for mrET patients.

## References

[1] Bhatia KP , Bain P , Bajaj N , Elble RJ , Hallett M , Louis ED , et al . Consensus statement on the classification of tremors. from the task force on tremor of the International Parkinson and movement disorder Society . *Mov Disord* 2018 ; **33** : 75 – 87 . doi: 10.1002/mds.27121 29193359 PMC6530552

[2] Busenbark KL , Nash J , Nash S , Hubble JP , Koller WC . Is essential tremor benign? *Neurology* 1991 ; **41** : 1982 – 83 . doi: 10.1212/wnl.41.12.1982 1745359

[3] Bain PG . Tremor assessment and quality of life measurements . *Neurology* 2000 ; **54** : S26 – 29 . 10854349

[4] Louis ED , Ottman R . How many people in the USA have essential tremor? deriving a population estimate based on epidemiological data . *Tremor Other Hyperkinet Mov (N Y)* 2014 ; **4** : 259 . doi: 10.7916/D8TT4P4B 25157323 PMC4137360

[5] Essential Tremor NEW [Internet] . Tremor | The National Tremor Foundation . 2021 . Available from : https://tremor.org.uk/essential-tremor ( accessed 4 Oct 2021 )

[6] Louis ED , Barnes L , Albert SM , Cote L , Schneier FR , Pullman SL , et al . Correlates of functional disability in essential tremor . *Mov Disord* 2001 ; **16** : 914 – 20 . doi: 10.1002/mds.1184 11746622

[7] Louis ED , Barnes LF , Wendt KJ , Albert SM , Pullman SL , Yu Q , et al . Validity and test-retest reliability of a disability questionnaire for essential tremor . *Mov Disord* 2000 ; **15** : 516 – 23 . doi: 10.1002/1531-8257(200005)15:3<516::AID-MDS1015>3.0.CO;2-J 10830418

[8] Madelein van der Stouwe AM , Nieuwhof F , Helmich RC . Tremor pathophysiology: lessons from neuroimaging . *Curr Opin Neurol* 2001 ; **33** : 474 – 81 . doi: 10.1097/WCO.0000000000000829 32657888

[9] Nicoletti V , Cecchi P , Pesaresi I , Frosini D , Cosottini M , Ceravolo R . Cerebello-thalamo-cortical network is intrinsically altered in essential tremor: evidence from a resting state functional MRI study . *Sci Rep* 2020 ; **10** ( 1 ): 16661 . doi: 10.1038/s41598-020-73714-9 33028912 PMC7541442

[10] Schnitzler A , Münks C , Butz M , Timmermann L , Gross J . Synchronized brain network associated with essential tremor as revealed by magnetoencephalography . *Mov Disord* 2009 ; **24** : 1629 – 35 . doi: 10.1002/mds.22633 19514010

[11] Shanker V . Essential tremor: diagnosis and management . *BMJ* 2019 ; **366** : l4485 . doi: 10.1136/bmj.l4485 31383632

[12] Dallapiazza RF , Lee DJ , De Vloo P , Fomenko A , Hamani C , Hodaie M , et al . Outcomes from stereotactic surgery for essential tremor . *J Neurol Neurosurg Psychiatry* 2019 ; **90** : 474 – 82 . doi: 10.1136/jnnp-2018-318240 30337440 PMC6581115

[13] Favilla CG , Ullman D , Wagle Shukla A , Foote KD , Jacobson CE , Okun MS . Worsening essential tremor following deep brain stimulation: disease progression versus tolerance . *Brain* 2012 ; **135** : 1455 – 62 . doi: 10.1093/brain/aws026 22344584

[14] Meng Y , Hynynen K , Lipsman N . Applications of focused ultrasound in the brain: from thermoablation to drug delivery . *Nat Rev Neurol* 2021 ; **17** : 7 – 22 . doi: 10.1038/s41582-020-00418-z 33106619

[15] Ter Haar G . Hifu tissue ablation: concept and devices . *Adv Exp Med Biol* 2016 ; **880** : 3 – 20 . doi: 10.1007/978-3-319-22536-4_1 26486329

[16] Elias WJ , Lipsman N , Ondo WG , Ghanouni P , Kim YG , Lee W , et al . A randomized trial of focused ultrasound thalamotomy for essential tremor . *N Engl J Med* 2016 ; **375** : 730 – 39 . doi: 10.1056/NEJMoa1600159 27557301

[17] Halpern CH , Santini V , Lipsman N , Lozano AM , Schwartz ML , Shah BB , et al . Three-Year follow-up of prospective trial of focused ultrasound thalamotomy for essential tremor . *Neurology* 2019 ; **93** : e2284 – 93 . doi: 10.1212/WNL.0000000000008561 31748250

[18] Jameel A , Gedroyc W , Nandi D , Jones B , Kirmi O , Molloy S , et al . Two year data from a preliminary study of double lesion site mrgfus treatment of essential tremor targeting the thalamus and the posterior subthalamic area . *MedRxiv Prepr* 2021 ; **2;2** . doi: https://doi.org/0.12.27.20248723 10.1080/02688697.2021.1958150 34382881

[19] Elias WJ , Huss D , Voss T , Loomba J , Khaled M , Zadicario E , et al . A pilot study of focused ultrasound thalamotomy for essential tremor . *N Engl J Med* 2013 ; **369** : 640 – 48 . doi: 10.1056/NEJMoa1300962 23944301

[20] National Institute for Health and Clinical Excellence . National Institute for Health and Clinical Excellence (November 2012) The guidelines manual [Internet]. London: NICE . 2012 . Available from : https://www.nice.org.uk/process/pmg6/chapter/assessing-cost-effectiveness ( accessed 8 Oct 2020 )

[21] O’Donnell JC , Pham SV , Pashos CL , Miller DW , Smith MD . Health technology assessment: lessons learned from around the world -- an overview . *Value Health* 2009 ; **12 Suppl 2** : S1 – 5 . doi: 10.1111/j.1524-4733.2009.00550.x 19523179

[22] Dakin H , Devlin N , Feng Y , Rice N , O’Neill P , Parkin D . The influence of cost-effectiveness and other factors on NICE decisions . *Health Econ* 2015 ; **24** : 1256 – 71 . doi: 10.1002/hec.3086 25251336

[23] Siebert U , Alagoz O , Bayoumi AM , Jahn B , Owens DK , Cohen DJ , et al . State-transition modeling: a report of the ISPOR-SMDM modeling good research practices Task force -- 3 . *Value Health* 2012 ; **15** : 812 – 20 . doi: 10.1016/j.jval.2012.06.014 22999130

[24] Li C , Gajic-Veljanoski O , Schaink AK , Higgins C , Fasano A , Sikich N , et al . Cost-Effectiveness of magnetic resonance-guided focused ultrasound for essential tremor . *Mov Disord* 2019 ; **34** : 735 – 43 . doi: 10.1002/mds.27587 30589951

[25] Health Quality Ontario . Magnetic resonance-guided focused ultrasound neurosurgery for essential tremor: a health technology assessment . *Ont Health Technol Assess Ser* 2018 ; **18** : 1 – 141 . PMC5963668 29805721

[26] Fahn S , Tolosa E , Marin C . *Clinical Rating Scale for Tremor* ; 1988 . , pp . 271 – 80 .

[27] Stacy MA , Elble RJ , Ondo WG , Wu S-C , Hulihan J , TRS study group . Assessment of interrater and intrarater reliability of the fahn-tolosa-marin tremor rating scale in essential tremor . *Mov Disord* 2007 ; **22** : 833 – 38 . doi: 10.1002/mds.21412 17343274

[28] Sinai A , Nassar M , Eran A , Constantinescu M , Zaaroor M , Sprecher E , et al . Magnetic resonance-guided focused ultrasound thalamotomy for essential tremor: a 5-year single-center experience . *J Neurosurg* 2019 ; 1 – 8 . doi: 10.3171/2019.3.JNS19466 31277064

[29] National Institute for Health and Clinical Excellence . National Institute for Health and ClinicalExcellence (November 2012) The guidelines manual [Internet]. London: NICE . 2012 . Available from : https://www.nice.org.uk/process/pmg6/chapter/assessing-cost-effectiveness ( accessed 8 Oct 2020 )

[30] Kim M , Jung NY , Park CK , Chang WS , Jung HH , Chang JW . Comparative evaluation of magnetic resonance-guided focused ultrasound surgery for essential tremor . *Stereotact Funct Neurosurg* 2017 ; **95** : 279 – 86 . doi: 10.1159/000478866 28810261

[31] Huss DS , Dallapiazza RF , Shah BB , Harrison MB , Diamond J , Elias WJ . Functional assessment and quality of life in essential tremor with bilateral or unilateral DBS and focused ultrasound thalamotomy . *Mov Disord* 2015 ; **30** : 1937 – 43 . doi: 10.1002/mds.26455 26769606

[32] Langford BE , Ridley CJA , Beale RC , Caseby SCL , Marsh WJ , Richard L . Focused ultrasound thalamotomy and other interventions for medication-refractory essential tremor: an indirect comparison of short-term impact on health-related quality of life . *Value Health* 2018 ; **21** : 1168 – 75 : S1098-3015(18)30299-7 . doi: 10.1016/j.jval.2018.03.015 30314617

[33] NHS England . National Cost Collection for the NHS: National schedule of NHS costs (2018/19) [Internet] . 2018 . Available from : https://www.england.nhs.uk/national-cost-collection/#ncc1819 ( accessed 3 Mar 2021 )

[34] Herceg M , Nagy F , Pál E , Janszky J , Késmárky I , Komoly S , et al . Pramipexole may be an effective treatment option in essential tremor . *Clin Neuropharmacol* 2012 ; **35** : 73 – 76 . doi: 10.1097/WNF.0b013e31824687bf 22318193

[35] NICE [Internet] . Judging whether public health interventions offer value for money | Advice . Available from : https://www.nice.org.uk/advice/lgb10 ( accessed 7 Oct 2021 )

[36] Favilla CG , Ullman D , Wagle Shukla A , Foote KD , Jacobson CE , Okun MS . Worsening essential tremor following deep brain stimulation: disease progression versus tolerance . *Brain* 2012 ; **135** : 1455 – 62 . doi: 10.1093/brain/aws026 22344584

[37] Dogu O , Sevim S , Camdeviren H , Sasmaz T , Bugdayci R , Aral M , et al . Prevalence of essential tremor: door-to-door neurologic exams in Mersin Province, Turkey . *Neurology* 2003 ; **61** : 1804 – 6 . doi: 10.1212/01.wnl.0000099075.19951.8c 14694055

[38] Office for National Statistics . National life tables: England - Period expectation of life based on data for the years 2017-2019 [Internet] . 2020 . Available from : https://www.ons.gov.uk/peoplepopulationandcommunity/birthsdeathsandmarriages/lifeexpectancies/datasets/nationallifetablesenglandreferencetables ( accessed 18 Jan 2021 )

[39] Louis ED , Ferreira JJ . How common is the most common adult movement disorder? update on the worldwide prevalence of essential tremor . *Mov Disord* 2010 ; **25** : 534 – 41 . doi: 10.1002/mds.22838 20175185

[40] Koller WC , Busenbark K , Miner K . The relationship of essential tremor to other movement disorders: report on 678 patients. essential tremor Study Group . *Ann Neurol* 1994 ; **35** : 717 – 23 . doi: 10.1002/ana.410350613 8210229

[41] Zesiewicz TA , Elble RJ , Louis ED , Gronseth GS , Ondo WG , Dewey RB , et al . Evidence-Based guideline update: treatment of essential tremor . *Neurology* 2011 ; **77** : 1752 – 55 . 22013182 10.1212/WNL.0b013e318236f0fd PMC3208950

[42] Jameel A , Gedroyc W , Nandi D , Jones B , Kirmi O , Molloy S , et al . Double lesion mrgfus treatment of essential tremor targeting the thalamus and posterior sub-thalamic area: preliminary study with two year follow-up . *Br J Neurosurg* 2022 ; **36** : 241 – 50 . doi: 10.1080/02688697.2021.1958150 34382881

[43] Elias WJ , Lipsman N , Ondo WG , Ghanouni P , Kim YG , Lee W , et al . A randomized trial of focused ultrasound thalamotomy for essential tremor . *N Engl J Med* 2016 ; **375** : 730 – 39 . doi: 10.1056/NEJMoa1600159 27557301

[44] Boston Scientific . Vercise GeviaTM Information for Prescribers [Internet] . 2019 . Available from : https://www.bostonscientific.com/content/dam/Manuals/us/current-rev-en/92152385-04_Vercise_Gevia%E2%84%A2_Information_for_Prescribers_en-US_s.pdf ( accessed 3 Mar 2021 )

[45] Igarashi A , Tanaka M , Abe K , Richard L , Peirce V , Yamada K . Cost-minimisation model of magnetic resonance-guided focussed ultrasound therapy compared to unilateral deep brain stimulation for essential tremor treatment in Japan . *PLOS ONE* 2019 ; **14** ( 7 ): e0219929 . doi: 10.1371/journal.pone.0219929 31314791 PMC6636755

[46] Ravikumar VK , Parker JJ , Hornbeck TS , Santini VE , Pauly KB , Wintermark M , et al . Cost-Effectiveness of focused ultrasound, radiosurgery, and DBS for essential tremor . *Mov Disord* 2017 ; **32** : 1165 – 73 . doi: 10.1002/mds.26997 28370272

[47] Igarashi A , Tanaka M , Abe K , Richard L , Peirce V , Yamada K . Cost-minimisation model of magnetic resonance-guided focussed ultrasound therapy compared to unilateral deep brain stimulation for essential tremor treatment in Japan . *PLOS ONE* 2019 ; **14** ( 7 ): e0219929 . doi: 10.1371/journal.pone.0219929 31314791 PMC6636755

[48] Health Quality Ontario . Magnetic resonance-guided focused ultrasound neurosurgery for essential tremor: a health technology assessment . *Ont Health Technol Assess Ser* 2018 ; **18** : 1 – 141 . PMC5963668 29805721

[49] Iorio-Morin C , Yamamoto K , Sarica C , Zemmar A , Levesque M , Brisebois S , et al . Bilateral focused ultrasound thalamotomy for essential tremor (BEST-FUS phase 2 trial) . *Mov Disord* 2021 ; **36** : 2653 – 62 . doi: 10.1002/mds.28716 34288097

[50] Fukutome K , Hirabayashi H , Osakada Y , Kuga Y , Ohnishi H . Bilateral magnetic resonance imaging-guided focused ultrasound thalamotomy for essential tremor . *Stereotact Funct Neurosurg* 2022 ; **100** : 44 – 52 . doi: 10.1159/000518662 34515233

[51] Martínez-Fernández R , Mahendran S , Pineda-Pardo JA , Imbach LL , Máñez-Miró JU , Büchele F , et al . Bilateral staged magnetic resonance-guided focused ultrasound thalamotomy for the treatment of essential tremor: a case series study . *J Neurol Neurosurg Psychiatry* 2021 ; **92** : 927 – 31 . doi: 10.1136/jnnp-2020-325278 33906933

