# Supplementary material for: The cost-effectiveness of unilateral magnetic resonance-guided focused ultrasound in comparison with unilateral deep brain stimulation for the treatment of medically refractory essential tremor in England
Source: Br J Radiol. 2022 Sep 27;95(1140):20220137. doi: 10.1259/bjr.20220137 (PMC9733625; doi:10.1259/bjr.20220137)
Supplement: Supplementary Table 1. [file bjr.20220137.suppl-01.docx]

# Appendices

Appendix 1 *– Model Approach*

Appendix 2 *- Model Results and further Analyses*

Appendix 3 *– Supplementary Tables*

Appendix 4 *– Supplementary Figures*

*Appendix References*

# Appendix 1 – Model Approach

**Model approach**

This cost-utility analysis has been adapted from a previously published Canadian model (1,2) and was conducted from the perspective of NHS England. A Markov cohort model was chosen as it accounts for different health states over time. The two comparator strategies (DBS and MRgFUS) had the same model structure and used a cycle length of 1 year.

A 5-year time horizon was used as no long-term data is available for MRgFUS past 5 years (3) and most of the expected differences between strategies would be captured within this time period (tremor recurrence and long-term adverse events). A discount rate of 3.5% was applied to costs and utilities in the model in line with NICE guidelines (4). The model was constructed in Microsoft Excel (Microsoft Corporation, Redmond, Washington DC, USA).

**Health states modelled**

Four health states were included in the model: “baseline tremor”, “mild to moderate improved tremor”, “marked improved tremor”, and “tremor recurrence”. The “baseline tremor” state contains those patients who continue to live with disabling ET if they undergo no procedure (DBS or MRgFUS) or have an unsuccessful procedure (a less than 10% improvement in their CRST score). The “mild to moderate improved tremor” state and “marked improved tremor” state includes those patients who have a 10-50% improvement and a 50-100% improvement in their CRST score, respectively (1,2). The “tremor recurrence” state is a temporary state where patients, who have already undergone a procedure, experience tremor recurrence that requires reoperation.

**Flow of patients between states**

As shown in Figure 1, all patients enter the model with medically refractory ET. Patients who have a procedure (i.e., MRgFUS or DBS depending on the strategy) undergo the procedure at the start of the model and, depending on their clinical outcomes, either remain in this state or enter the “marked improved tremor” or “mild to moderate improved tremor” health state. From the “marked improved tremor” and “mild to moderate improved tremor” health states some patients move to the “tremor recurrence” state. In the next model cycle, these patients move back to baseline tremor if they have an unsuccessful procedure or do not have a reoperation. If they have a successful second procedure, they move to either “marked improved tremor” or “mild to moderate improved tremor”. Over time, a proportion of patients move from the “marked improved tremor” to mild to moderate improved tremor” health state, i.e., the benefits from the procedure wane.

**Adverse events**

Patients who have undergone a procedure might experience adverse events (i.e., side effects) that affect their quality of life and/or incur healthcare costs. Adverse effects were only included if they required physician input or had a large impact on patient health-related quality of life (Appendix 3 Supplementary Table S2). These may be "transient” adverse effects (i.e., short-term events that happen within a year after surgery and then disappear) or “permanent” adverse effects (i.e., long term events that persist for longer than 1 year after the procedure was undertaken). For example, a proportion of patients who undergo an MRgFUS procedure may experience gait disturbance or chorea that resolve within a few months (transient adverse effects) (5). However, in some patients gait disturbance or paraesthesia can persist and therefore become permanent adverse events. DBS adverse events were classified into procedure-related (6–8) (i.e., infection and intracranial haemorrhage), hardware-related (6,9,10) (i.e., lead fracture), and stimulation related (11,12) (i.e., gait disturbance or speech problems) adverse effects. Intracranial haemorrhage is a procedure-related adverse event and therefore only occurs in the first 12-months post-procedure whereas the other adverse events can happen over the entire model time horizon.

**Mortality**

At any point during the model time frame, a patient may die from all-cause mortality. It is assumed that there is no additional excess mortality from either ET or the procedures (DBS or MRgFUS) (5).

**Outcomes**

The model outcomes were the average total costs and quality-adjusted life-years (QALYs) for each strategy over the time period. The incremental cost-effectiveness ratio (ICER) of MRgFUS versus DBS and MRgFUS versus no procedure was then determined.

**Probability inputs**

Although some inputs and assumptions have been taken from the model developed by Li et al., 2018, (1,2) as they are still applicable to ET in England, this model does use additonal data to make it more representative. The key model parameters are shown in Table 1.

***Improvement post-procedure:*** As there are no head-to-head studies of MRgFUS versus DBS, no direct comparison of clinical effectiveness can be made. Looking at data from retrospective studies for DBS and MRgFUS (13,14), as well as a recent systematic literature review comparing DBS and MRgFUS (15) it was assumed that DBS and MRgFUS have the same clinical effectiveness but with different adverse event profiles. The onset of treatment benefit of MRgFUS was assumed to be immediate, whereas the benefit for DBS was assumed to start at 3 months due to the time it takes to program and optimise the device settings (validated by clinical experts).

The proportions of patients with marked or mild to moderate improvement after MRgFUS were based on results from a small clinical trial in England ((16)). Unlike Li et al., in this model, it was assumed that patients who experienced less than 10% improvement did not experience tremor recurrence. Instead, these patients stayed in the baseline state after an unsuccessful procedure. In the England clinical trial data, 12/13 (92.3%) and 1/13 (7.7%) patients experienced marked improvement and mild to moderate improvement post-procedure respectively. Due to the small sample size and zero observations in the no improvement outcome option, the Wilson method was used to augment the data. Five extra cases were added to the total group denominator. Four cases were deemed a ‘success’ and moved to the marked improvement health state. One case was deemed a ‘failure’ and was split in a 1:4 ratio between the proportion of patients with no improvement (one previous case) and those with mild to moderate improvement (previously zero cases) leaving 88.9% (16/18) patients with marked improvement, 10.0% (1.8/18) with mild to moderate improvement, and 1.1% (0.2/18) with no improvement post-procedure in the base case (17,18). The values used by Li et al., were used for scenario analysis.

***Tremor recurrence:*** Although ET progresses slowly and patients’ symptoms will deteriorate over time (19), all patients in the model were assumed to have stable disease; patients only deteriorated if they had tremor recurrence or due to waning surgical effectiveness. According to Favilla et al., (20), the probability of tremor recurrence (defined in the study as a pattern of tolerance) following DBS is 4% (1/28) from 6-36 months and Cruz et al demonstrating exponential increase reliance on electrical stimulation from 2 years post procedure (21). For MRgFUS the probability of recurrence (returning to baseline tremor) at 2 years post-procedure was assumed to be 8.3% (1/12 – one patient dropped out of the study) based on data from the England clinical trial. The study rates were then converted to an annual probability in the model assuming a constant probability over time.

***Reoperation after tremor recurrence:*** Unlike Li et al. (1,2), we assumed that 5% of people who undergo MRgFUS and experience tremor recurrence will then undergo reoperation and receive the same surgical procedure as their first procedure. However, as in Li et al., we assumed that reoperation resulted in the same outcomes as the primary procedure.

**Cost inputs**

The costs were estimated from the perspective of NHS England and were reported in 2019 GBP. We included the following costs: surgical procedure, medication for ET, ongoing patient monitoring, and management of adverse effects. The DBS procedure cost was estimated using NHS reimbursement costs (22) and validated by clinical experts (Appendix 3 Supplementary Table S3). The cost included all pre-, peri- and post-procedure and follow-up costs for the first year. For MRgFUS, the NHS reimbursement cost was provided by Imperial College Healthcare NHS Trust included all pre-, peri-, post-procedure and follow-up costs for the first 5 years. The model assumes there are no costs associated with the transient or permanent adverse events for MRgFUS as they are managed during normal follow-up. All patients were assumed to have the same ET medications costs independent of which health state they were in. Costing details can be found in the Appendix 3 Supplementary Tables S3-7.

**Utilities**

Following Li et al., we used utility values from Herceg et al., 2012 (23) for patients in the “baseline tremor” and “marked improved tremor” health states. The utility for the “mild to moderate tremor improvement” health state was assumed to be an average between the “baseline tremor” and “marked improved tremor” health state utilities. It was assumed that the utility of “tremor recurrence” was the same as that of “baseline tremor”.

To include decreases in quality of life due to the transient and permanent adverse events, a weighted disutility was calculated. First, the disutility assigned to each adverse event was calculated by using the difference between the utility reported in the literature (24–30) and the average utility of a healthy person from England (31). These were then time-adjusted by the average duration of each adverse event to calculate the disutility per one year. Next, each disutility was weighted by the proportion of patients who experience the adverse event. The utility for each health state was then calculated by taking away the weighted disutility. There was no disutility associated with reoperation and any adverse events were captured in the same manner as for the first procedure. For the deterministic sensitivity analysis (DSA) the low value indicates the ‘worst case’ i.e., the highest proportion of adverse events with the lowest associated utility value and the high value indicates the ‘best case’ i.e., the lowest proportion of adverse events with the highest associated utility value. More details can be found in the Appendix 3: Supplementary Tables S8-9.

# Appendix 2 - model results and further analyses

***Scenario Analyses***

Scenario analyses allow the model to be run under altered parameters to assess the cost-effectiveness of MRgFUS in mrET for various population parameters and behaviours. The results were sensitive to various assumptions regarding the time horizon, population parameters such as age, input parameters such as eligibility for DBS or proportion of patients in each health states post-MRgFUS, and various utilities used. A few examples of scenario analyses were performed assess the impact of changing parameters on the base case.

- Age: When the starting age of patients entering the population was varied, compared to DBS, the ICER remained dominant but there was little change in the incremental QALY (4). Compared to no procedure, the ICER reduced from £20,851 from the base case (aged 70) to £19,816 (aged 40).
- Time Horizon: The results were sensitive to the model time horizon (Appendix 3 Supplementary Table S10). When a 1-year and 10-year time horizon were used MRgFUS was still the dominant strategy compared to DBS in the base case. Compared to no procedure, the ICER in the base case was £84,539 and £13,940 at the 1- and 10-year time horizon respectively.
- Population eligibility for DBS: The current care scenario showed that MRgFUS was always a cost-effective option, but it became the dominant strategy if at least 30% of the population was eligible for DBS (Table 4).
- MRgFUS inputs - When the MRgFUS inputs were taken fom the 2018 Randomised Control trial (32) as used in the Canadian model, rather than the English clinical trial as used in the base case, the model produced similar results with MRgFUS being the dominant strategy compared to DBS and an ICER of £27,959 per QALY compared to no procedure (Table 3). The model with the RCT data was also run with several scenarios and the results were sensitive to various assumptions. (Appendix 3: Supplementary Table S11 and S12).

***Deterministic sensitivity analysis (DSA)***

A deterministic sensitivity analysis (DSA) was conducted to determine which parameters had the largest effect on the model outcomes. The probabilities, utilities and costs in the model were varied between their upper and lower limits (either the 95% confidence interval (CI) from baseline or by 20% of the original values (Appendix 3 Supplementary Table S9 and S10). For DSA the low value indicates the ‘worst case’ i.e., the highest proportion of adverse events with the lowest associated utility value and the high value indicates the ‘best case’ i.e., the lowest proportion of adverse events with the highest associated utility value. More details can be found in the Appendix 3 Supplementary Tables S8-S9.

The impact on the ICER is presented in a tornado graph, (Figure 2) showing the difference between the MRgFUS and DBS strategies. Only those parameters that had more than a 5% difference, either using the high or low value, from the base case value were reported. A few examples are described below:

ICER for MRgFUS versus no procedure: When the parameters were varied between their low and high values, these assumptions had the biggest impact (Figure 2):

- Base-line utility (£12,517- 62,397) suggesting that MRgFUS may not be as cost-effective in those patients who are not as affected by the disease
- The cost of MRgFUS procedure (£16,535- 25,167)
- Utility for the marked improvement health state in year 2 onwards for those undergoing MRgFUS (£25,504- 18,691)

DBS vs. MRgFUS: When the parameters were varied between their low and high values, these assumptions had the biggest impact:

- Utility for the marked improvement health state in the first year for those undergoing MRgFUS (-£20,561,429 to -£697,086),
- Utility for baseline state in the first year for those undergoing DBS (-£672,348 to -£13,748,994)
- The probability of recurrence for those undergoing DBS (-£4,720,374 to -£418,743) ICER for MRgFUS versus no procedure: When the parameters were varied between their low and high values, these assumptions had the biggest impact (Appendix 4 Supplementary Figure F2).
- Base-line utility (£12,517- 62,397)
- The cost of MRgFUS procedure (£16,535- 25,167)
- Utility for the marked improvement health state in year 2 onwards for those undergoing MRgFUS (£25,504- 18,691)

***Probabilistic sensitivity analysis (PSA)***

A Monte Carlo PSA was run with 1,000 iterations to explore the robustness of the ICER. Every input value was independently sampled from their assigned distribution (based on data from the literature or an estimated distribution). Probabilities were assigned a beta distribution and cost parameters were assigned a gamma distribution (Appendix 3 Supplementary Tables S9 and S10). All inputs shared by the two strategies (MRgFUS and DBS) were assigned the same value for that given iteration; all other inputs were varied independently between the strategies. The impact on the outcomes of varying the parameter inputs in the PSA is shown in Figure 3. A few examples are described below:

- Compared to no procedure the MRgFUS strategy was more cost-effective in 66% of the 1,000 iterations and in under 2% of iterations were the differences in QALYs negative. Base case had a mean ICER of £22,268).
- For MRgFUS versus DBS the distribution reveals the uncertainty in the results with an average ICER of -£1,032,614 per QALY (range -£175,779 to £934,874).
- The probability of MRgFUS being cost-effective at different Willingness to Pay Thresholds (WTP) (i.e., different maximum values for an acceptable cost per gained QALY) was reported as a cost-effectiveness acceptability curve.

# Appendix 3: Supplementary Tables

[Table S1 Epidemiological inputs used to derive the estimated patient population in England eligible for MRgFUS procedure 2](#_Toc79357015)

[Table S2 Treatment-related adverse effects and complications included in the model 3](#_Toc79357016)

[Table S3 Average cost of DBS surgical procedure (2019 £) 4](#_Toc79357017)

[Table S4 Average cost of DBS battery replacement procedure (2019 £) 6](#_Toc79357018)

[Table S5 Annual cost for ongoing medication (2019 £) 7](#_Toc79357019)

[Table S6 Annual monitoring costs (2019 £) 8](#_Toc79357020)

[Table S7 Average costs of DBS adverse effects (2019 £) 9](#_Toc79357021)

[Table S8 Distributions for probabilities, costs, utilities and other input parameters used in the base case in the probabilistic and deterministic sensitivity analysis 10](#_Toc79357022)

[Table S9 Distributions for probabilities parameters used in the RCT scenario in the probabilistic and deterministic sensitivity analysis 13](#_Toc79357023)

[Table S10 The results for the outcomes (MRgFUS versus all comparators); 1-year horizon and 10-year horizon 14](#_Toc79357024)

[Table S11 The results for the outcomes (MRgFUS versus all comparators); Age scenario (RCT data) 15](#_Toc79357025)

[Table S12 The results for the outcomes (assuming 100% of patients are suitable for MRgFUS versus a certain % of patients are not suitable for DBS); Current care scenario (RCT data) 16](#_Toc79357026)

Table S1 Epidemiological inputs used to derive the estimated patient population in England eligible for MRgFUS procedure

| **Parameter** | **Assumption** | **Baseline value** | **Source** |
| --- | --- | --- | --- |
| Estimated England population aged 70 |  | 569,617 | (33) |
| Estimated prevalence of essential tremor among those aged ≥ 65 years | 4.6% | 26,202 | (34) |
| Proportion seeking medical care | 45% | 11,791 | Assumption (based on expert opinion) |
| Proportion with moderate to severe disease | 30% | 3,537 | Assumption taken from (1) |
| Proportion medication refractory or intolerant | 50% | 1,769 | (35,36); Assumption (based on expert opinion) |
| Proportion eligible for surgery (without drug contraindications) | 80% | 1,415 | Assumption (based on expert opinion) |

ONS, Office for National Statistics

Table S2 Treatment-related adverse effects and complications included in the model

| **Treatment** | **Side effects or complications** | **Proportion of patients** | | **Source** | **Average duration** | **Source** |
| --- | --- | --- | --- | --- | --- | --- |
| MRgFUS |  | Base case | RCT |  |  |  |
| 1-year post-surgery: | |  |  |  |  |  |
|  | Gait disturbance | 15.3% | 12.5% | Base case: (37)  RCT scenario (2,5): | 2.8 months | (2,5) |
|  | Speech problem* | 0% | 3.6% |  | 6 months | (2,5) |
|  | Headache | 0% | 3.6% |  | 1.8 months | (2,5) |
| Permanent: | |  |  |  |  |  |
|  | Gait disturbance | 7.7% | 8.9% |  | Permanent | (2,5) |
|  | Chorea | 7.7% | 0.0% |  | Permanent | (37) |
| DBS |  |  |  |  |  |  |
| 1-year post-surgery (procedure-related): | |  |  |  |  |  |
|  | Infection | 1.8% | Same as base case | (10) | 1 month | (7); expert opinion |
|  | Intracranial haemorrhage | 1.6% |  | (10) | 1 month | (38) |
| Hardware-related: | |  |  |  |  |  |
|  | Lead fracture or migration | 1.5% |  | (10) | 1 month | Assume same as infection |
| Stimulation-related: | |  |  |  |  |  |
|  | Gait disturbance | 8.1% |  | (39) | Permanent | (38,39) |
|  | Speech problem* | 12.3% |  | (12) | Permanent |  |

MRgFUS, magnetic resonance guided focused ultrasound; DBS, deep brain stimulation

*Speech problem includes side effects such as hypophonia, dysarthria, aphasia/dysphasia, and dysphagia.

Table S3 Average cost of DBS surgical procedure (2019 £)

| **Resource Item** | | **Cost (£)** | **Data source and comments** |
| --- | --- | --- | --- |
| Pre-procedure | |  |  |
|  | MRI | 136 | NHS Reference Costs 2018-2019, RD01A (Diagnostic imaging, Magnetic Resonance Imaging scan of one area, without contrast, 19 years and over, outpatient) (22) |
|  | CT | 83 | NHS Reference Costs 2018-2019 RD20A (Diagnostic imaging, Computerised Tomography scan of one area, without contrast, 19 years and over, outpatient) (22) |
|  | Psychological tests | 479 | NHS Reference Costs 2018-2019, AA32Z (Neurosurgery, neuropsychology tests, outpatient procedures) (22) |
|  | Anaesthesiologist first appointment | 183 | NHS Reference Costs 2016-2017, WF01B (Consultant led non-admitted face-to-face attendance, first, anaesthetics) (22) |
|  | Radiologist/neurologist/neurosurgeon joint first appointment | 245 | NHS Reference Costs 2018-2019, WF02B (Consultant-led multiprofessional non-admitted face-to-face attendance, first, neurology) (22) |
|  | Psychologist first appointment | 315 | NHS Reference Costs 2018-2019, WF01B (Consultant led non-admitted face-to-face attendance, first, clinical psychology) (22) |
| **Total pre-procedure costs** | | **1,441** |  |
| Peri-Procedure | |  |  |
|  | Surgery | 7,715 | NHS Reference Cost 2018-2019 for AA60A (Insertion of neurostimulator for treatment of neurological conditions, 19 years and over, elective inpatient) (22) |
|  | Implantable pulse generator | 19,800 | Expert opinion; Boston Vercise Gevia model (40) |
|  | Electrode | 9,888 |  |
|  | Extension lead | 1,730 |  |
|  | Patient controller/therapy controller | 1,145 |  |
|  | Travel case | 37 |  |
|  | Charging kit | 1,980 |  |
| **Total pre-procedure costs** | | **42,296** |  |
| Peri-Procedure | |  |  |
|  | Neurologist follow-up appointment (5 units) | 844 | NHS Reference Costs 2018-2019, WF01A (Consultant led non-admitted face-to-face attendance, follow-up, neurology) (22) |
|  | Day case admission | 2,665 | NHS Reference Cost 2018-2019 for AA60A (Insertion of neurostimulator for treatment of neurological conditions, 19 years and over, day case) |
|  | Psychologist follow-up appointment | 298 | NHS Reference Costs 2018-2019, WF01A (Consultant led non-admitted face-to-face attendance, follow-up, clinical psychology) |
|  | CT | 83 | NHS Reference Costs 2018-2019 RD20A (Diagnostic imaging, CT scan of one area, without contrast, 19 years and over, outpatient) |
| **Total peri-procedure costs** | | **3,890** |  |
| **Total DBS costs** | | **47,627** |  |

DBS, deep brain stimulation

This is operational cost only; capital cost (equipment, installation, and maintenance costs) is excluded in the base case analysis.

This includes all resource use for year 1 after surgery.

Table S4 Average cost of DBS battery replacement procedure (2019 £)

| **Resource Item** |  | **Cost (£)** | **Data source and comments** |
| --- | --- | --- | --- |
| Peri-Procedure |  |  |  |
|  | Surgery | 7,715 | NHS Reference Cost 2018-2019 for AA60A (Insertion of neurostimulator for treatment of neurological conditions, 19 years and over, elective inpatient) (22) |
|  | Implantable pulse generator | 19,800 | Expert opinion; Boston Vercise Gevia model (40) |
| **Total pre-procedure costs** | | **27,515** |  |
| Peri-Procedure |  |  |  |
|  | Neurologist follow-up appointment | 169 | NHS Reference Costs 2018-2019, WF01A (Consultant led non-admitted face-to-face attendance, follow-up, neurology) (22) |
| **Total peri-procedure costs** | | **169** |  |
| **Total DBS battery replacement costs** | | **27,684** |  |

DBS, deep brain stimulation

Table S5 Annual cost for ongoing medication (2019 £)

| **Resource Item** | **Average dose (mg/day)** | **Patients using drug (%)** | **Annual cost (£)** |
| --- | --- | --- | --- |
| Propranolol | 180 | 75 | 174 |
| Primidone | 350 | 25 | 461 |
| Topiramate | 300 | 25 | 80 |
| Alprazolam | 1.5 | 25 | 29 |
| **Total ongoing medication costs** | |  | **744** |

The average dose for each drug was sourced from clinical experts. Annual costs came from the British National Formulary (41) using a weighted average by formulation from dispensing data provided the NHS Prescription Cost Analysis (PCA) data (42)

Table S6 Annual monitoring costs (2019 £)

| **Resource Item** |  | **Annual cost (£)** | **Data source and comments** |
| --- | --- | --- | --- |
| No procedure | |  |  |
|  | Neurologist follow-up appointment | 169 | NHS Reference Costs 2018-2019, WF01A (Consultant led non-admitted face-to-face attendance, follow-up, neurology) (22) |
| **Total no procedure annual monitoring costs** | | **169** |  |
| MRgFUS | |  |  |
|  | Neurologist follow-up appointment | 169 | NHS Reference Costs 2018-2019, WF01A (Consultant led non-admitted face-to-face attendance, follow-up, neurology) (22) |
| **Total MRgFUS annual monitoring costs*** | | **169** |  |
| DBS |  |  |  |
|  | Neurologist follow-up appointment (3 attendances) | 507 | NHS Reference Costs 2018-2019, WF01A (Consultant led non-admitted face-to-face attendance, follow-up, neurology) (22) |
|  | Day case admission | 2,665 | NHS Reference Cost 2018-2019 for AA60A (Insertion of neurostimulator for treatment of neurological conditions, 19 years and over, day case) (22) |
| **Total DBS annual monitoring costs*** | | **3,172** |  |

MRgFUS, magnetic resonance guided focused ultrasound; DBS, deep brain stimulation

*This applies from year 5 for MRgFUS, as the procedure cost covers the first 5 years of follow-up whereas DBS monitoring applies from year 2

Table S7 Average costs of DBS adverse effects (2019 £)

| **Resource Item** | | **Cost (£)** | **Resource used** | **Data source and comments** |
| --- | --- | --- | --- | --- |
| Adverse events requiring physician input | | |  |  |
|  | Infection | 657 | 80% treated as outpatients (flucloxacillin 2g four times a day for 4 weeks);20% would have a wound washout (of the pectoral housing pocket) | Average dose sourced from clinical experts; drug costs (41) weighted by average dispensed formulation (42);  NHS Reference Cost 2018-2019 for AA60A (Insertion of neurostimulator for treatment of neurological conditions, 19 years and over, day case) (22) |
|  | Intracranial haemorrhage | 20,545 | Hospitalisation for symptomatic haemorrhage | Average of NHS Reference Costs 2018-2019, non-elective long stay, AA50A Very Complex Intracranial Procedures, 19 years and over, with CC 0-12+ (22) |
| DBS hardware-related complications | |  |  |  |
|  | Lead fracture or migration | 14,777 | Reoperation to replace the lead | Same cost as primary DBS cost in Table S3, but the only hardware cost included is the lead |
| DBS simulation-related complications | | |  |  |
|  | Gait disturbance | 0 | No treatment required | Assumption (based on expert opinion) |
|  | Speech problem | 169 | 1 follow-up neurologist appointment every year | One repeat neurologist visit per unit; NHS Reference Costs 2018-2019, WF01A (Consultant led non-admitted face-to-face attendance, follow-up, neurology) (22) |

MRgFUS, magnetic resonance guided focused ultrasound; DBS, deep brain stimulation; PCA, Prescription Cost Analysis
There are no costs associated with the short- or long-term adverse events for MRgFUS as the costs are included in the 5-year MRgFUS costs and then are managed during normal follow-up

Table S8 Distributions for probabilities, costs, utilities and other input parameters used in the base case in the probabilistic and deterministic sensitivity analysis

| **Parameter** | | **Distribution** | **Baseline value** | **DSA value (Low)** | **DSA value (High)** | **Alpha*** | **Beta*** | **Source (for baseline value)** |
| --- | --- | --- | --- | --- | --- | --- | --- | --- |
| Probabilities | |  |  |  |  |  |  |  |
|  | Proportion with marked (5-100%) improvement post-surgery  (DBS & MRgFUS equally effective) | Beta | 1.1% | 6% | 0% | 16.0 | 2.0 | (37) |
|  | Proportion with mild to moderate (10-50%) improvement post-surgery  (DBS & MRgFUS equally effective) | Beta | 10.0% | 23% | 0% | Varies with marked proportion (1-marked improvement) in the same ratio with no improvement as the baseline value | | |
|  | Proportion with no (<10%) improvement post-surgery  (DBS & MRgFUS equally effective) | Beta | 88.9% | 71% | 100% | Varies with marked proportion (1-marked improvement) in the same ratio with mild improvement as the baseline value | | |
|  | Proportion (annually) moving from marked improvement to mild due to a wane in surgical effectiveness (MRgFUS) | Beta | 7.7% | 0.0% | 22.2% | 1.0 | 13.0 | (37) |
|  | Proportion (annually) moving from marked improvement to mild due to a wane in surgical effectiveness (DBS) | Beta | 9.2% | 0.0% | 20.5% | 348.7 | 3443.7 | (20) |
|  | Annual probability of recurrence (DBS) | Beta | 1.4% | 0.0% | 5.7% | 15.1 | 1032.5 | (20) |
|  | Annual probability of recurrence (MRgFUS) | Beta | 1.0% | 0.8% | 1.2% | 380.2 | 36874.4 | (37) |
|  | Probability of reoperation after recurrence (MRgFUS) | Beta | 5.0% | 4.0% | 6.0% | 364.9 | 6933.1 | Assumption (based on expert opinion) |
| Utilities | |  |  |  |  |  |  |  |
|  | Normal healthy person in England | Beta | 0.85 | 0.68 | 1.02 | 55.6 | 9.6 | (31) |
|  | Alive with disabling tremor (baseline) | Beta | 0.69 | 0.57 | 0.81 | 16.6 | 7.4 | (23) |
|  | Improved tremor post-surgery (marked improvement – no adverse events) | Beta | 0.91 | 0.88 | 0.94 | 21.8 | 2.2 | (23) |
|  | Improved tremor post-surgery (mild to moderate improvement – no adverse events) | Beta | 0.80 | 0.72 | 0.88 | - | - | Assumption |
|  | Gait disturbance | Beta | 0.82 | 0.78 | 0.86 | 69.7 | 15.3 | (24) |
|  | Speech disorder | Beta | 0.54 | 0.50 | 0.58 | 63.2 | 53.8 | (26) |
|  | Infection | Beta | 0.64 | 0.54 | 0.74 | 14.8 | 8.2 | (27,28) |
|  | Intracranial haemorrhage | Beta | 0.60 | 0.02 | 1.00 | 36.0 | 24.0 | (29) |
|  | Lead fracture or migration | Beta | 0.66 | 0.53 | 0.79 | 2296.8 | 1183.2 | (30) |
| Adverse events - MRgFUS | | | | | | | | |
|  | Gait disturbance (year 1) | Beta | 15.4% | 0% | 35% | 2.0 | 11.0 | (37) |
|  | Speech disorders (year 1) | Beta | 0.0% | 0% | 0% | 0.0 | 13.0 | (37) |
|  | Chorea (year 1) | Beta | 15.4% | 0% | 35% | 2.0 | 11.0 | (37) |
|  | Gait disturbance (year 2+) | Beta | 7.7% | 0% | 22% | 1.0 | 12.0 | (37) |
|  | Chorea (year 2+) | Beta | 7.7% | 0% | 22% | 1.0 | 12.0 | (37) |
| Adverse events - DBS | | | | | | | | |
|  | Infection | Uniform | 1.8% | 0.8% | 2.7% | 13.0 | 715.0 | (10) |
|  | Intracranial haemorrhage | Uniform | 1.6% | 0.7% | 2.6% | 12.0 | 716.0 | (10) |
|  | Lead fracture or migration | Uniform | 1.8% | 0.8% | 2.7% | 12.0 | 716.0 | (10) |
|  | Gait disturbance | Beta | 9.1% | 0% | 21% | 2.0 | 20.00 | (39) |
|  | Speech disorders | Beta | 12.3% | 8% | 19% | 26.0 | 188.6 | (12) |
| Costs | |  |  |  |  |  |  |  |
|  | MRgFUS surgical procedure (all year 1-5 resource use) | Gamma | £16,500 | £13,200.00 | 19,800.00 | 25.0 | 660.0 | Assumption (based on expert opinion) |
|  | DBS surgical procedure (all year 1 resource use) | Gamma | £47,627 | £38,101 | £57,152 | 25.0 | 1905.1 | See Table S3 |
|  | Battery replacement surgery | Gamma | £27,684 | £22,634 | £33,221 | 25.0 | 1107.4 | Expert opinion |
|  | Annual medication costs | Gamma | £743.80 | £595.04 | £892.56 | 25.0 | 29.8 | See Table S5 |
|  | Annual monitoring costs – MRgFUS | Gamma | £169 | £135 | £203 | 25.0 | 6.8 | See Table S6 |
|  | Annual monitoring costs - DBS | Gamma | £3,172 | £2,537 | £3,806 | 25.0 | 126.9 | See Table S6 |
|  | Annual monitoring costs – No procedure | Gamma | £169 | £135 | £203 | 25.0 | 6.8 | See Table S6 |
| Others | |  |  |  |  |  |  |  |
|  | Discount rate (costs and QALYs) | - | 3.5% | 1.5% | 5% | - | - | (4) |
|  | Age (years) | Normal | 70 | 56 | 84 | - | - | (5) |
|  | Proportion of males in England | Beta | 49% | 40% | 59% | 193.74 | 198.14 | ONS |
|  |  |  |  |  |  |  |  |  |
|  |  |  |  |  |  |  |  |  |
|  | DBS onset of benefit (months) | Normal | 3.00 | 0.00 | 3.60 | - | - | Assumption (based on expert opinion) |
|  | DBS battery life (years) | Normal | 15.00 | 5.00 | 20.00 | - | - | Assumption (based on expert opinion); Boston Vercise Gevia model (40) |
|  | Year after which wane in surgical effectiveness starts e.g. moves from marked to mild/mod (DBS) | Normal | 2.00 | 1.00 | 5.00 |  |  | Assumption (based on expert opinion) |
|  | Year after which wane in surgical effectiveness starts e.g. moves from marked to mild/mod (MRgFUS) | Normal | 2.00 | 1.00 | 5.00 |  |  | Assumption (based on expert opinion) |

*For values that were assigned a gamma distribution the alpha and beta values were estimated using alpha = mean^2/variance and beta = variance/mean. DSA high and low values for values assigned a gamma distribution varied the value +/- 20%. For values that were assigned a beta distribution the sample size (N) and the number of patients with that event (n), if available, were used to estimate the alpha (n) and beta (N-n) values, otherwise, alpha and beta were estimated using the method of moments if sample means and standard errors were reported instead. DSA high and low values for values with beta distributions used the 95% confidence intervals that were found within the referenced base case study or that were calculated.
MRgFUS, magnetic resonance guided focused ultrasound; DBS, deep brain stimulation; ONS, Office for National Statistics

Table S9 Distributions for probabilities parameters used in the RCT scenario in the probabilistic and deterministic sensitivity analysis

| **Parameter** | | **Distribution** | **Baseline value** | **DSA value (Low)** | **DSA value (High)** | **Alpha** | **Beta** | | **Source** | |
| --- | --- | --- | --- | --- | --- | --- | --- | --- | --- | --- |
| Probabilities | |  |  |  |  |  |  | |  | |
|  | Proportion with marked (5-100%) improvement post-surgery  (DBS & MRgFUS equally effective) | Beta | 48% | 46% | 50% | 27 | 29 | | (2,5) | |
|  | Proportion with mild to moderate (10-50%) improvement post-surgery  (DBS & MRgFUS equally effective) | Beta | 43% | 44% | 41% | Varies with marked proportion (1-marked improvement) in the same ratio with no improvement as the baseline value | | | | |
|  | Proportion with no (<10%) improvement post-surgery  (DBS & MRgFUS equally effective) | Beta | 9% | 9% | 9% | Varies with marked proportion (1-marked improvement) in the same ratio with mild improvement as the baseline value | | | | |
| Adverse events - MRgFUS | | | | | | | | | | |
|  | Gait disturbance (year 1) | Beta | 12.5% | 3.8% | 21.2% | 7.0 | | 49.0 | | (2,5) |
|  | Speech disorders (year 1) | Beta | 3.6% | 0.0% | 8.4% | 2.0 | | 54.0 | | (2,5) |
|  | Chorea (year 1) | Beta | 0.0% | 0% | 0% | - | | - | | (2,5) |
|  | Gait disturbance (year 2+) | Beta | 8.9% | 1.5% | 16.4% | 5.00 | | 51.00 | | (2,5) |
|  | Chorea (year 2+) | Beta | 0.0% | 0% | 0% | - | | - | | (2,5) |

*For values that were assigned a gamma distribution the alpha and beta values were estimated using alpha = mean^2/variance and beta = variance/mean. DSA high and low values for values assigned a gamma distribution varied the value +/- 20%. For values that were assigned a beta distribution the sample size (N) and the number of patients with that event (n), if available, were used to estimate the alpha (n) and beta (N-n) values, otherwise, alpha and beta were estimated using the method of moments if sample means and standard errors were reported instead. DSA high and low values for values with beta distributions used the 95% confidence intervals that were found within the referenced base case study or that were calculated.
MRgFUS, magnetic resonance guided focused ultrasound; DBS, deep brain stimulation;

Table S10 The results for the outcomes (MRgFUS versus all comparators); 1-year horizon and 10-year horizon

| **Strategy** | **Costs (£)** | **Δ Cost (£)** | **QALY** | **Δ QALY** | **ICER (£/QALY)** |
| --- | --- | --- | --- | --- | --- |
| **1-year time horizon** | | | | | |
| Base case (England data) | | | | | |
| MRgFUS | 24,346,577 |  | 1,204 |  |  |
| No procedure | 1,000,255 | 23,346,322 | 928 | 276 | **84,539** |
| DBS | 69,159,791 | -44,813,214 | 1,135 | 69 | **Dominant** |
|  | | | | | |
| MRgFUS | 24,346,577 |  | 1,125 |  |  |
| No procedure | 1,000,255 | 23,346,322 | 928 | 197 | **118,372** |
| DBS | 69,159,791 | -44,813,214 | 1,081 | 44 | **Dominant** |
| **10-year time horizon** | | | | | |
| Base case (England data) | | | | | |
| MRgFUS | 32,103,966 |  | 8,831 |  |  |
| No procedure | 9,289,156 | 22,814,810 | 7,195 | 1,637 | **13,940** |
| DBS | 105,319,422 | -73,215,456 | 8,799 | 32 | **Dominant** |
| Scenario based on RCT data | | | | | |
| MRgFUS | 32,087,364 |  | 8,438 |  |  |
| No procedure | 9,289,156 | 22,798,209 | 7,195 | 1,244 | **18,332** |
| DBS | 103,120,211 | -71,032,847 | 8,284 | 155 | **Dominant** |

Difference and total values are reported to the nearest integer.

DBS, deep brain stimulation; MRgFUS, magnetic resonance-guided focused ultrasound; ICER, incremental cost-effectiveness ratio

Table S11 The results for the outcomes (MRgFUS versus all comparators); Age scenario (RCT data)

| **Age** |  | **Costs (£)** | **Δ Cost (£)** | **QALY** | **Δ QALY** | **ICER (£/QALY)** |
| --- | --- | --- | --- | --- | --- | --- |
| 60 | MRgFUS | 28,136,144 |  | 5,148 |  |  |
|  | No procedure | 5,471,828 | 22,664,316 | 4,310 | 838 | **27,040** |
|  | DBS | 87,816,254 | -59,680,109 | 5,037 | 111 | **Dominant** |
| 50 | MRgFUS | 28,199,814 |  | 5,216 |  |  |
|  | No procedure | 5,546,355 | 22,653,459 | 4,367 | 849 | **26,690** |
|  | DBS | 88,117,209 | -59,917,395 | 5,104 | 113 | **Dominant** |
| 40 | MRgFUS | 28,225,279 |  | 5,243 |  |  |
|  | No procedure | 5,576,134 | 22,649,145 | 4,390 | 853 | **26,552** |
|  | DBS | 88,237,126 | -60,011,846 | 5,130 | 113 | **Dominant** |

Difference and total values are reported to the nearest integer.

DBS, deep brain stimulation; MRgFUS, magnetic resonance-guided focused ultrasound; ICER, incremental cost-effectiveness ratio

Table S12 The results for the outcomes (assuming 100% of patients are suitable for MRgFUS versus a certain % of patients are not suitable for DBS); Current care scenario (RCT data)

| **Strategy** | **Patients eligible for DBS (%)** | **Costs (£)** | **Δ Cost (£)** | **QALY** | **Δ QALY** | **ICER (£/QALY)** |
| --- | --- | --- | --- | --- | --- | --- |
| MRgFUS |  | 27,976,355 |  | 4,979 |  |  |
| Current care | 0 | 5,284,832 | 22,691,523 | 4,167 | 812 | **27,959** |
|  | 10 | 19,421,239 | 8,555,116 | 4,262 | 717 | **11,933** |
|  | 20 | 21,905,935 | 6,070,420 | 4,280 | 699 | **8,683** |
|  | 30 | 30,166,664 | -2,190,308 | 4,341 | 638 | **Dominant** |
|  | 40 | 38,394,177 | -10,417,822 | 4,406 | 573 | **Dominant** |
|  | 50 | 46,588,476 | -18,612,121 | 4,475 | 504 | **Dominant** |
|  | 60 | 54,749,561 | -26,773,205 | 4,547 | 432 | **Dominant** |
|  | 70 | 62,877,430 | -34,901,074 | 4,623 | 356 | **Dominant** |
|  | 80 | 70,972,084 | -42,995,729 | 4,702 | 277 | **Dominant** |
|  | 90 | 79,033,523 | -51,057,168 | 4,784 | 195 | **Dominant** |
|  | 100 | 87,061,748 | -59,085,393 | 4,871 | 108 | **Dominant** |

Current care is a blend of patients who have no procedure and those that undergo the DBS procedure. Difference and total values are reported to the nearest integer.
DBS, deep brain stimulation; MRgFUS, magnetic resonance-guided focused ultrasound; ICER, incremental cost-effectiveness ratio

# Appendix 4: Supplementary Figures

[Figure S1 Results of the deterministic sensitivity analysis for the RCT scenario for A) MRgFUS vs no procedure B) MRgFUS vs DBS: the impact on the ICER of varying each parameter individually to the high and low values in a one-way sensitivity analysis 17](#_Toc79357027)

[Figure S2 Result of probabilistic analysis for the RCT scenario for MRgFUS vs no procedure and MRgFUS vs DBS: based on 1,000 iterations illustrating the distribution of the ICERs 19](#_Toc79357028)

[Figure S3 Cost-effectiveness acceptability curve using the RCT data for MRgFUS vs No Procedure and MRgFUS vs DBS: showing the probability that MRgFUS is cost-effective at different Willingness to Pay Thresholds for cost-effectiveness 20](#_Toc79357029)

Figure S1 Results of the deterministic sensitivity analysis for the RCT scenario for A) MRgFUS vs no procedure B) MRgFUS vs DBS: the impact on the ICER of varying each parameter individually to the high and low values in a one-way sensitivity analysis

| A | 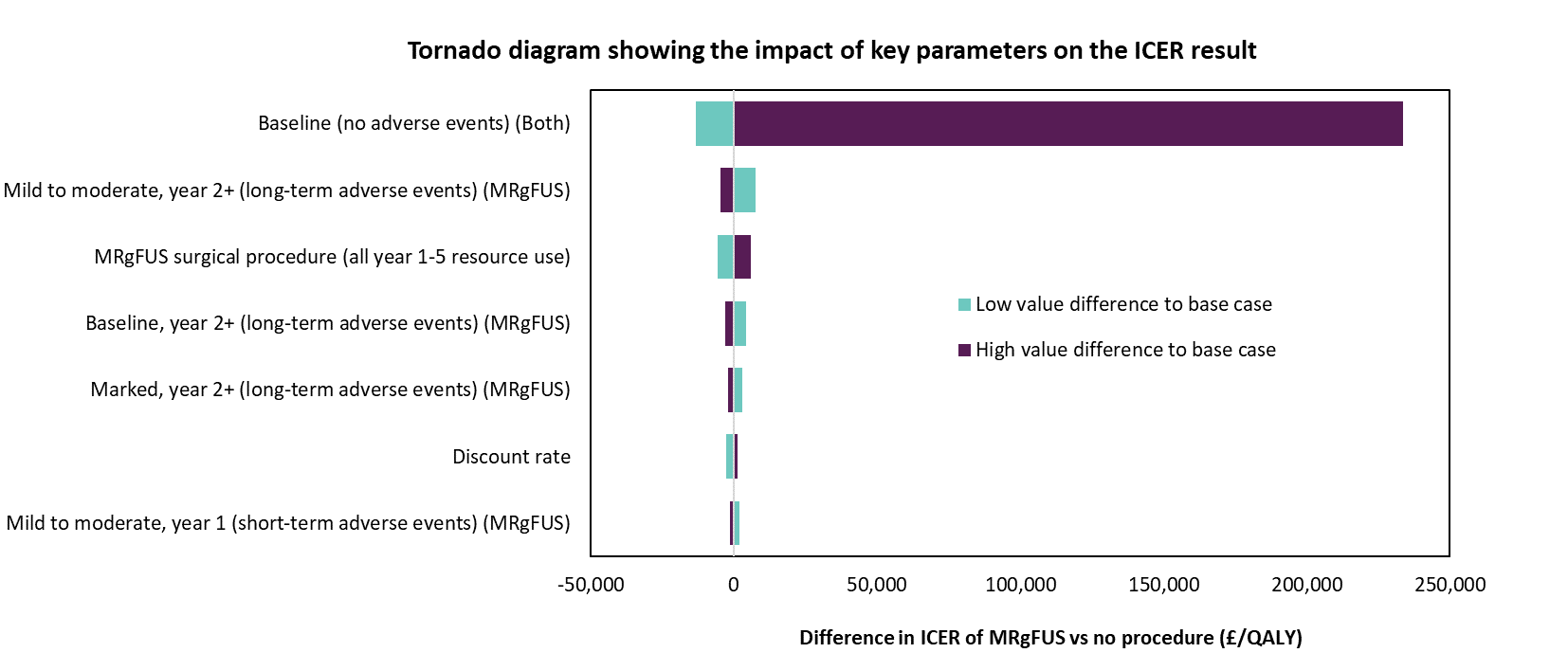 |
| --- | --- |

| B | 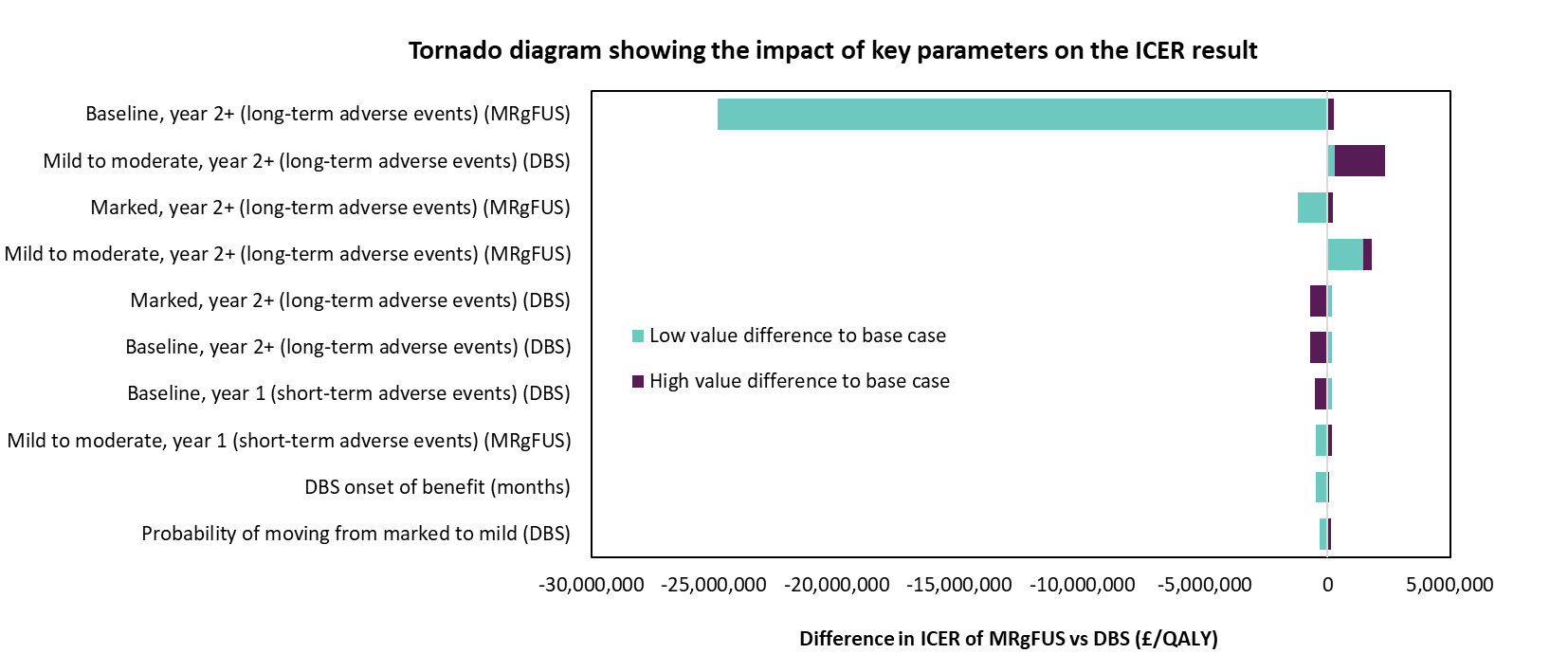 |
| --- | --- |

DBS, deep brain stimulation; MRgFUS, magnetic resonance-guided focused ultrasound; ICER, incremental cost-effectiveness ratio

Figure S2 Result of probabilistic analysis for the RCT scenario for MRgFUS vs no procedure and MRgFUS vs DBS: based on 1,000 iterations illustrating the distribution of the ICERs


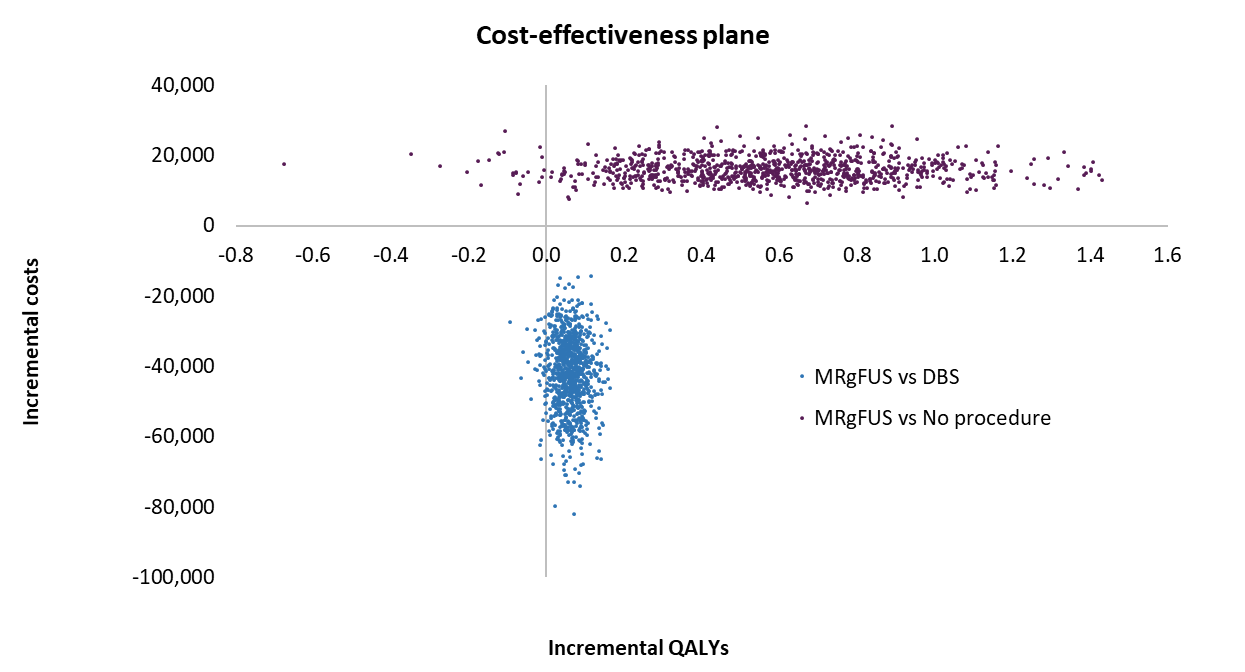


DBS, deep brain stimulation; MRgFUS, magnetic resonance-guided focused ultrasound; ICER, incremental cost-effectiveness ratio; QALYs, quality-adjusted life years

Figure S3 Cost-effectiveness acceptability curve using the RCT data for MRgFUS vs No Procedure and MRgFUS vs DBS: showing the probability that MRgFUS is cost-effective at different Willingness to Pay Thresholds for cost-effectiveness


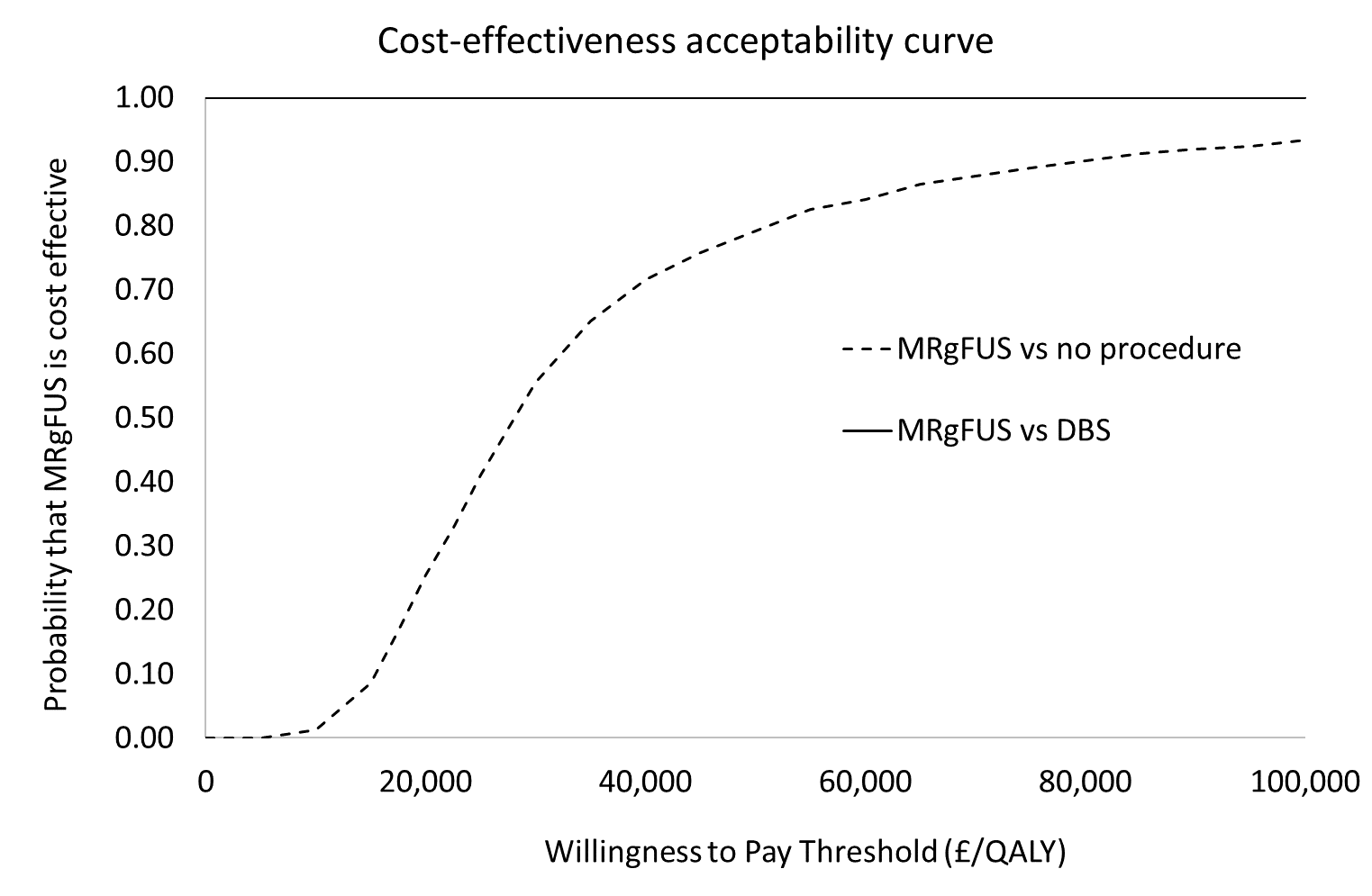
DBS, deep brain stimulation; MRgFUS, magnetic resonance-guided focused ultrasound; ICER, incremental cost-effectiveness ratio; QALYs, quality-adjusted life years

# REFERENCES for the Appendix

1. Li C, Gajic-Veljanoski O, Schaink AK, Higgins C, Fasano A, Sikich N, et al. Cost-Effectiveness of Magnetic Resonance-Guided Focused Ultrasound for Essential Tremor. Mov Disord Off J Mov Disord Soc. 2019;34(5):735–43.

2. Health Quality Ontario. Magnetic Resonance-Guided Focused Ultrasound Neurosurgery for Essential Tremor: A Health Technology Assessment. Ont Health Technol Assess Ser. 2018;18(4):1–141.

3. Sinai A, Nassar M, Eran A, Constantinescu M, Zaaroor M, Sprecher E, et al. Magnetic resonance-guided focused ultrasound thalamotomy for essential tremor: a 5-year single-center experience. J Neurosurg. 2019 Jul 5;1–8.

4. National Institute for Health and Clinical Excellence. National Institute for Health and Clinical Excellence (November 2012) The guidelines manual [Internet]. London: NICE; [cited 2020 Oct 8]. Available from: https://www.nice.org.uk/process/pmg6/chapter/assessing-cost-effectiveness

5. Elias WJ, Lipsman N, Ondo WG, Ghanouni P, Kim YG, Lee W, et al. A Randomized Trial of Focused Ultrasound Thalamotomy for Essential Tremor. N Engl J Med. 2016 Aug 25;375(8):730–9.

6. Fishman PS. Thalamotomy for essential tremor: FDA approval brings brain treatment with FUS to the clinic. 2017 Jul 13 [cited 2021 Mar 4]; Available from: https://www.ncbi.nlm.nih.gov/pmc/articles/PMC5508673/

7. Bjerknes S, Skogseid IM, Sæhle T, Dietrichs E, Toft M. Surgical Site Infections after Deep Brain Stimulation Surgery: Frequency, Characteristics and Management in a 10-Year Period. PLOS ONE. 2014 Aug 14;9(8):e105288.

8. Sillay KA, Larson PS, Starr PA. Deep brain stimulator hardware-related infections: incidence and management in a large series. Neurosurgery. 2008 Feb;62(2):360–6; discussion 366-367.

9. Mohammed N, Patra D, Nanda A. A meta-analysis of outcomes and complications of magnetic resonance–guided focused ultrasound in the treatment of essential tremor. Neurosurg Focus. 2018 Feb;44(2):E4.

10. Fenoy AJ, Simpson RK. Risks of common complications in deep brain stimulation surgery: management and avoidance. J Neurosurg. 2014 Jan;120(1):132–9.

11. Flora ED, Perera CL, Cameron AL, Maddern GJ. Deep brain stimulation for essential tremor: A systematic review. Mov Disord. 2010;25(11):1550–9.

12. Alomar S, King NKK, Tam J, Bari AA, Hamani C, Lozano AM. Speech and language adverse effects after thalamotomy and deep brain stimulation in patients with movement disorders: A meta-analysis. Mov Disord Off J Mov Disord Soc. 2017;32(1):53–63.

13. Kim M, Jung NY, Park CK, Chang WS, Jung HH, Chang JW. Comparative Evaluation of Magnetic Resonance-Guided Focused Ultrasound Surgery for Essential Tremor. Stereotact Funct Neurosurg. 2017;95(4):279–86.

14. Huss DS, Dallapiazza RF, Shah BB, Harrison MB, Diamond J, Elias WJ. Functional assessment and quality of life in essential tremor with bilateral or unilateral DBS and focused ultrasound thalamotomy. Mov Disord Off J Mov Disord Soc. 2015 Dec;30(14):1937–43.

15. Langford BE, Ridley CJA, Beale RC, Caseby SCL, Marsh WJ, Richard L. Focused Ultrasound Thalamotomy and Other Interventions for Medication-Refractory Essential Tremor: An Indirect Comparison of Short-Term Impact on Health-Related Quality of Life. Value Health. 2018 Oct 1;21(10):1168–75.

16. Jameel A, Gedroyc W, Nandi D, Jones B, Kirmi O, Molloy S, et al. Double lesion MRgFUS treatment of essential tremor targeting the thalamus and posterior sub-thalamic area: preliminary study with two year follow-up. Br J Neurosurg. 2021 Aug 12;1–10.

17. Wilson EB. Probable Inference, the Law of Succession, and Statistical Inference. J Am Stat Assoc. 1927 Jun 1;22(158):209–12.

18. Lewis J, Sauro J. When 100% Really Isn’t 100%: Improving the Accuracy of Small-Sample Estimates of Completion RatesJUS. 2006 May 7 [cited 2021 Jul 16]; Available from: https://uxpajournal.org/when-100-really-isnt-100-improving-the-accuracy-of-small-sample-estimates-of-completion-rates/

19. Louis ED, Michalec M, Gillman A. Shaky drawing: what is the rate of decline during prospective follow-up of essential tremor? BMJ Open. 2014 Apr 10;4(4):e004626.

20. Favilla CG, Ullman D, Wagle Shukla A, Foote KD, Jacobson CE IV, Okun MS. Worsening essential tremor following deep brain stimulation: disease progression versus tolerance. Brain. 2012 May 1;135(5):1455–62.

21. Rodríguez Cruz PM, Vargas A, Fernández-Carballal C, Garbizu J, De La Casa-Fages B, Grandas F. Long-term Thalamic Deep Brain Stimulation for Essential Tremor: Clinical Outcome and Stimulation Parameters. Mov Disord Clin Pract. 2016 Nov;3(6):567–72.

22. NHS England. National Cost Collection for the NHS: National schedule of NHS costs (2018/19) [Internet]. 2018 [cited 2021 Mar 3]. Available from: https://www.england.nhs.uk/national-cost-collection/#ncc1819

23. Herceg M, Nagy F, Pál E, Janszky J, Késmárky I, Komoly S, et al. Pramipexole May Be an Effective Treatment Option in Essential Tremor. Clin Neuropharmacol. 2012 Apr;35(2):73–6.

24. Yardley L, Barker F, Muller I, Turner D, Kirby S, Mullee M, et al. Clinical and cost effectiveness of booklet based vestibular rehabilitation for chronic dizziness in primary care: single blind, parallel group, pragmatic, randomised controlled trial. BMJ. 2012 Jun 6;344:e2237.

25. Thoma A, Wong VH, Sprague S, Duku E. A cost-utility analysis of open and endoscopic carpal tunnel release. Can J Plast Surg J Can Chir Plast. 2006;14(1):15–20.

26. Tengs TO, Wallace A. One thousand health-related quality-of-life estimates. Med Care. 2000 Jun;38(6):583–637.

27. Lee BY, Wiringa AE, Bailey RR, Goyal V, Tsui B, Lewis GJ, et al. The Economic Effect of Screening Orthopedic Surgery Patients Preoperatively for Methicillin-Resistant Staphylococcus aureus. Infect Control Hosp Epidemiol. 2010 Nov;31(11):1130–8.

28. Gheorghe A, Moran G, Duffy H, Roberts T, Pinkney T, Calvert M. Health Utility Values Associated with Surgical Site Infection: A Systematic Review. Value Health J Int Soc Pharmacoeconomics Outcomes Res. 2015 Dec;18(8):1126–37.

29. Lenert LA, Soetikno RM. Automated computer interviews to elicit utilities: potential applications in the treatment of deep venous thrombosis. J Am Med Inform Assoc JAMIA. 1997 Feb;4(1):49–56.

30. Gada H, Desai MY, Marwick TH. Cost-effectiveness of computed tomographic angiography before reoperative coronary artery bypass grafting: a decision-analytic model. Circ Cardiovasc Qual Outcomes. 2012 Sep 1;5(5):705–10.

31. Janssen MF, Szende A, Cabases J, Ramos-Goñi JM, Vilagut G, König HH. Population norms for the EQ-5D-3L: a cross-country analysis of population surveys for 20 countries. Eur J Health Econ HEPAC Health Econ Prev Care. 2019 Mar;20(2):205–16.

32. Elias WJ, Lipsman N, Ondo WG, Ghanouni P, Kim YG, Lee W, et al. A Randomized Trial of Focused Ultrasound Thalamotomy for Essential Tremor. N Engl J Med. 2016 Aug 25;375(8):730–9.

33. Office for National Statistics. National life tables: England - Period expectation of life based on data for the years 2017-2019 [Internet]. 2020 [cited 2021 Jan 18]. Available from: https://www.ons.gov.uk/peoplepopulationandcommunity/birthsdeathsandmarriages/lifeexpectancies/datasets/nationallifetablesenglandreferencetables

34. Louis ED, Ferreira JJ. How common is the most common adult movement disorder? Update on the worldwide prevalence of essential tremor. Mov Disord. 2010 Apr 15;25(5):534–41.

35. Koller WC, Busenbark K, Miner K. The relationship of essential tremor to other movement disorders: Report on 678 patients. Ann Neurol. 1994;35(6):717–23.

36. Zesiewicz TA, Elble RJ, Louis ED, Gronseth GS, Ondo WG, Dewey RB, et al. Evidence-based guideline update: Treatment of essential tremor. Neurology. 2011 Nov 8;77(19):1752–5.

37. Jameel A, Gedroyc W, Nandi D, Jones B, Kirmi O, Molloy S, et al. Two year data from a preliminary study of double lesion site MRgFUS treatment of Essential Tremor targeting the thalamus and the posterior subthalamic area. MedRxiv Prepr. 2021 Jan 2;2020.12.27.20248723.

38. Pahwa R, Lyons KE, Wilkinson SB, Tröster AI, Overman J, Kieltyka J, et al. Comparison of thalamotomy to deep brain stimulation of the thalamus in essential tremor. Mov Disord Off J Mov Disord Soc. 2001 Jan;16(1):140–3.

39. Peng-Chen Z, Morishita T, Vaillancourt D, Favilla C, Foote KD, Okun MS, et al. Unilateral thalamic deep brain stimulation in essential tremor demonstrates long-term ipsilateral effects. Parkinsonism Relat Disord. 2013 Dec;19(12):1113–7.

40. Boston Scientific. Vercise Gevia^TM^ Information for Prescribers [Internet]. 2019 [cited 2021 Mar 3]. Report No.: 92152385–04. Available from: https://www.bostonscientific.com/content/dam/Manuals/us/current-rev-en/92152385-04_Vercise_Gevia%E2%84%A2_Information_for_Prescribers_en-US_s.pdf

41. National Institute for Health and Care Excellence. British National Formulary (BNF) [Internet]. NICE; [cited 2021 Mar 2]. Available from: https://bnf.nice.org.uk/

42. NHS: Business Services Authority. Prescription Cost Analysis (PCA) data (August 2020) [Internet]. 2020 [cited 2021 Mar 3]. Available from: https://www.nhsbsa.nhs.uk/prescription-data/dispensing-data/prescription-cost-analysis-pca-data
